# The land snail fauna of Batu Kudik, isolated limestone outcrop near Simunjan, Sarawak, Malaysian Borneo (Mollusca, Gastropoda)

**DOI:** 10.3897/BDJ.12.e115556

**Published:** 2024-02-16

**Authors:** Jie Ying Lee, Nurul Syafiqah Nasir, Mohammad Effendi Marzuki, Jaap J. Vermeulen, Mohd Zacaery Khalik

**Affiliations:** 1 Faculty of Resource Science and Technology, Universiti Malaysia Sarawak, 94300, Kota Samarahan, Sarawak, Malaysia Faculty of Resource Science and Technology, Universiti Malaysia Sarawak, 94300 Kota Samarahan, Sarawak Malaysia; 2 Institute of Biodiversity and Environmental Conservation, Universiti Malaysia Sarawak, 94300, Kota Samarahan, Sarawak, Malaysia Institute of Biodiversity and Environmental Conservation, Universiti Malaysia Sarawak, 94300 Kota Samarahan, Sarawak Malaysia; 3 JK art and science, Lauwerbes 8 2318 AT, Leiden, Netherlands JK art and science, Lauwerbes 8 2318 AT Leiden Netherlands

**Keywords:** land snails, limestone, Sarawak, species abundance

## Abstract

**Background:**

The present study provides a checklist of land snails collected from Batu Kudik, a small and isolated limestone outcrop in Simunjan, Sarawak. A total of 24 species of land snails, representing 18 genera and 14 families were recorded, including one newly-described subspecies. The most species-rich of the families in Batu Kudik are Diplommatinidae (17%) and Chronidae (17%) with four recorded species from each of the families. Based on our analysis, *Plectostomawallaceikudikense* subsp. nov., *Opisthostomajavanica* and *Georissapyrrhoderma* were identified as the most abundant land snails at this isolated outcrop, whereas *Diplommatinaonyx* and *Everettiaminuta* were recorded as the least abundant. All of the land snails at Batu Kudik were exclusively found sheltered between limestone boulders, underscoring the critical role of this outcrop as their refuge for survival. Consequently, conserving this biodiversity-rich limestone area becomes paramount to prevent the local extinction of these land snail species and possibly other organisms that depend on the unique attributes of the limestone for their survival. We also provide detailed descriptions of *Plectostomawallaceikudikense*, a new subspecies of the genus *Plectostoma* which is endemic to Batu Kudik.

**New information:**

A description of a new subspecies *Plectostomawallaceikudikense* subsp. nov.

## Introduction

Karst areas (areas on limestone bedrock) of Borneo are well-known for their rich and varied biodiversity (Clement et al. 2006), including site-endemic and local-endemic species (Vermeulen and Whitten 1999). Liew et al. (2021) has charted the majority of all known limestone outcrops in Sabah and Sarawak (Malaysian Borneo) to facilitate land-use planning that minimises the impact of limestone quarrying.

Batu Kudik (1°12'35.38"N, 110°51'38.23"E) (see Fig. 1) is a cluster of two small limestone outcrops which are 5835 m^2^ and 497 m^2^, respectively. It is an isolated limestone outcrop which is similar to Bukit Sarang. Unfortunately, Batu Kudik limestone outcrops are not documented in the recent publication of Liew et al. (2021). Therefore, it has come to our attention after the publication of Vermeulen and Junau (2007) and Liew et al. (2021). We investigated its biodiversity making use of land snails (Mollusca Gastropoda) as an indicator group, since the recent publications have provided overviews of the local fauna (Vermeulen and Junau 2007, Marzuki et al. 2021, Vermeulen and Liew 2022) and, also, the land snails are often considered as limestone-bound endemic organisms.

**Figure 2.**
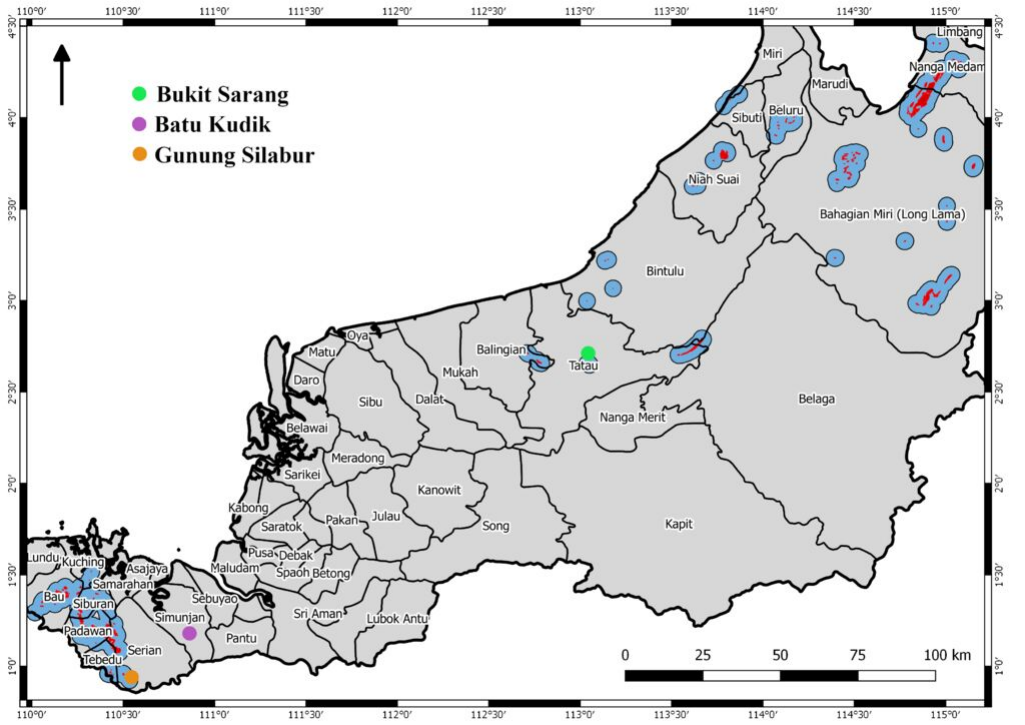
Limestone outcrops in the State of Sarawak, Malaysian Borneo. The red polygon represents the individual limestone outcrops, while the blue polygons around the limestone outcrop are background to emphasise outcrops that are too small to be seen on the map. The map is adapted from Liew et al. (2021). The green-coloured dot signifies the limestone outcrops of Bukit Sarang, the purple dot represents the limestone outcrops of Batu Kudik and the orange dot indicates the Gunung Silabur limestone outcrops.

Batu Kudik is of interest because it is approximately 49 km distant from the nearest limestone outcrop, Gunung Silabur and such spatial isolation is a driver of speciation amongst limestone-bound fauna. Additionally, Batu Kudik lies in between the limestone ranges south of Kuching (to the West) and Bukit Sarang (to the East) (see Fig. 2). Marzuki et al. (2021) found that the land snail fauna of a small part of the Kuching ranges (south of Bau) includes 47 out of 122 (38.5%) species, endemic to the Kuching ranges; the fauna of Bukit Sarang includes 26 out of ca. 83 (31.3%) species, endemic to the hill.

Therefore, this study presents the first checklist of the faunistic composition of land snails at Batu Kudik. Through this study, we elucidated the species richness and species abundance of the surrounding area. In addition, we describe a new subspecies namely, *Plectostomawallaceikudikense*.

## Materials and methods

Two separate surveys were conducted in October 2021 and April 2022 at Batu Kudik. The surveys were conducted around the limestone outcrops by a team of four individuals, with each survey taking a duration of at least four hours. Batu Kudik limestone outcrop is surrounded by an oil-palm plantation. During the surveys, live and empty shells were searched and collected, which consisted of sifting through leaf litter, scanning rock and wood surfaces and the surrounding karst vegetation. Top soils and leaf litters were collected to extract snails and empty shells via floatation methods. Then, shells were extracted from soil samples by manually picking up the shells and identifying them under a stereomicroscope. All specimens were identified to species level and the collected materials were deposited at Universiti Malaysia Sarawak (MZU.MOL) and the private collection (ME) of the third author. The identification of the species was based on the original description or a more recent publication of the respective species. The shell size classification were based on Vermeulen and Whitten (1998), which indicates the size of the microsnails are less than 5 mm, medium-sized snails range between 5 and 20 mm and the large snails are more than 20 mm in size.

Then, representatives of each of the species were photographed using a modified digital camera. For the setup and photoshoot, Capture One 15.0.0 was used for stack imaging. After that, the images were uploaded to Helicon 8.2.0 to create a composite image of the representative shell. The final images were scaled and cropped by using Adobe Photoshop 24.1 before importing into GIMP 2.10.32 to remove the background, sharpened and the images arranged onto a plate. The shell surfaces of representative paratype of *Plectostomawallaceikudikense* were coated with platinum for detailed examination in scanning electron microscope (SEM). For the analysis, species abundance was computed using RStudio (Team 2015) with R version 3.3.0. Species abundance percentage was calculated by dividing the number of individuals of a particular species by the total number of individuals and then converting the result to a percentage.

## Taxon treatments

### Plectostoma wallacei kudikense

Lee, Nasir, Marzuki, Vermeulen & Khalik, 2023 n.

8D13C012-1F29-5EC3-9F59-9492E7C2F422

34426B1B-897E-43B6-BEF9-FA15D9C5B546

#### Materials

- **Type status:** Holotype. **Occurrence:** catalogNumber: MZU.MOL.21.17; individualCount: 1; occurrenceID: 23E34535-FC8A-593B-942E-A4366A7F872C; **Taxon:** family: Diplommatinidae; genus: Plectostoma; scientificNameAuthorship: Lee, Nasir, Marzuki, Vermeulen & Khalik, 2023; **Location:** country: Malaysia, Sarawak; stateProvince: Samarahan; locality: Batu Kudik, small isolated limestone outcrop near Sungai Simunjan Kiri, approx. 14 miles SE Simunjan
- **Type status:** Paratype. **Occurrence:** catalogNumber: MZU.MOL.22.05; individualCount: 18; occurrenceID: 87105945-FEFE-53DE-A2C7-BDF2C6134ED9; **Taxon:** family: Diplommatinidae; genus: Plectostoma; scientificNameAuthorship: Lee, Nasir, Marzuki, Vermeulen & Khalik, 2023; **Location:** country: Malaysia, Sarawak; stateProvince: Samarahan; locality: Batu Kudik, small isolated limestone outcrop near Sungai Simunjan Kiri, approx. 14 miles SE Simunjan
- **Type status:** Paratype. **Occurrence:** catalogNumber: ME 13360; individualCount: 13; occurrenceID: 97B4820E-0338-5337-83C4-050FE8F15112; **Taxon:** family: Diplommatinidae; genus: Plectostoma; scientificNameAuthorship: Lee, Nasir, Marzuki, Vermeulen & Khalik, 2023; **Location:** country: Malaysia, Sarawak; stateProvince: Samarahan; locality: Batu Kudik, small isolated limestone outcrop near Sungai Simunjan Kiri, approx. 14 miles SE Simunjan
- **Type status:** Paratype. **Occurrence:** catalogNumber: ME 13895; individualCount: 23; occurrenceID: 1942E36B-DD50-5CB5-817F-E4C206B5B5C1; **Taxon:** family: Diplommatinidae; genus: Plectostoma; scientificNameAuthorship: Lee, Nasir, Marzuki, Vermeulen & Khalik, 2023; **Location:** country: Malaysia, Sarawak; stateProvince: Samarahan; locality: Batu Kudik, small isolated limestone outcrop near Sungai Simunjan Kiri, approx. 14 miles SE Simunjan
- **Type status:** Other material. **Occurrence:** catalogNumber: MZU.MOL.21.20; individualCount: 1773; occurrenceID: EA7B72FA-229B-5865-ADCB-C420F3C288B7; **Taxon:** family: Diplommatinidae; genus: Plectostoma; scientificNameAuthorship: Lee, Nasir, Marzuki, Vermeulen & Khalik, 2023; **Location:** country: Malaysia, Sarawak; stateProvince: Samarahan; locality: Batu Kudik, small isolated limestone outcrop near Sungai Simunjan Kiri, approx. 14 miles SE Simunjan

#### Description

Shell: spire conical with slightly convex sides. Apex is not or slightly oblique. Whorls 6 ½, convex; last whorl rounded or slightly angular at the periphery. Tuba free from the spire, abruptly narrowed towards the constriction, rounded below. Teleoconch: radial ribs on the spire are rather closely spaced (6 ribs/0.5 mm on penultimate whorl), slightly sinuous, often with a shallowly concave projection halfway, abrading to a not or slightly sinuous scar; those on the tuba widely spaced (6-12 ribs/0.5 mm half-way), not or hardly sinuous below (Fig. 3). Spiral striation present, distinct. Aperture: hardly tilted with regard to the coiling axis, circular to elliptic peristome simple or inconspicuous double, distant from the spire; outer peristome hardly spreading beyond the inner; inner peristome not protruding from the outer, widely spreading. Umbilicus: open, narrow, deep, 0.14-0.17 mm across. Dimensions: spire height 2.39-2.63 mm; spire width 1.49-1.64 mm, shell width (including tuba) 2.49-2.99 mm; aperture height 1.19-1.37 mm and aperture width 1.05-1.29 mm. Holotype dimensions: spire height 2.55 mm; spire width 1.57 mm, shell width (including tuba) 2.89 mm; aperture height 1.37 mm and aperture width 1.29 mm.

#### Diagnosis

*Plectostomawallaceikudikense* (Figs 4, 5), is different from the type subspecies together with other two known subspecies by having a shell with tuba free from the spire, simple or inconspicuous double peristome and constriction with a transverse palatalis, an oblique palatalis and a knob-shaped parietalis without longitudinal palatalis. Interestingly, the oblique palatalis is absent in most Bornean diplommatinids species and observed only in *Plectostomawallaceikudikense* and *Moussoniaisseli* (Godwin-Austen 1889).

#### Etymology

The specific epithet *kudikense* is in reference to the type locality, Batu Kudik.

#### Distribution

Known to be only endemic to Batu Kudik.

#### Ecology

**Figure 6.**
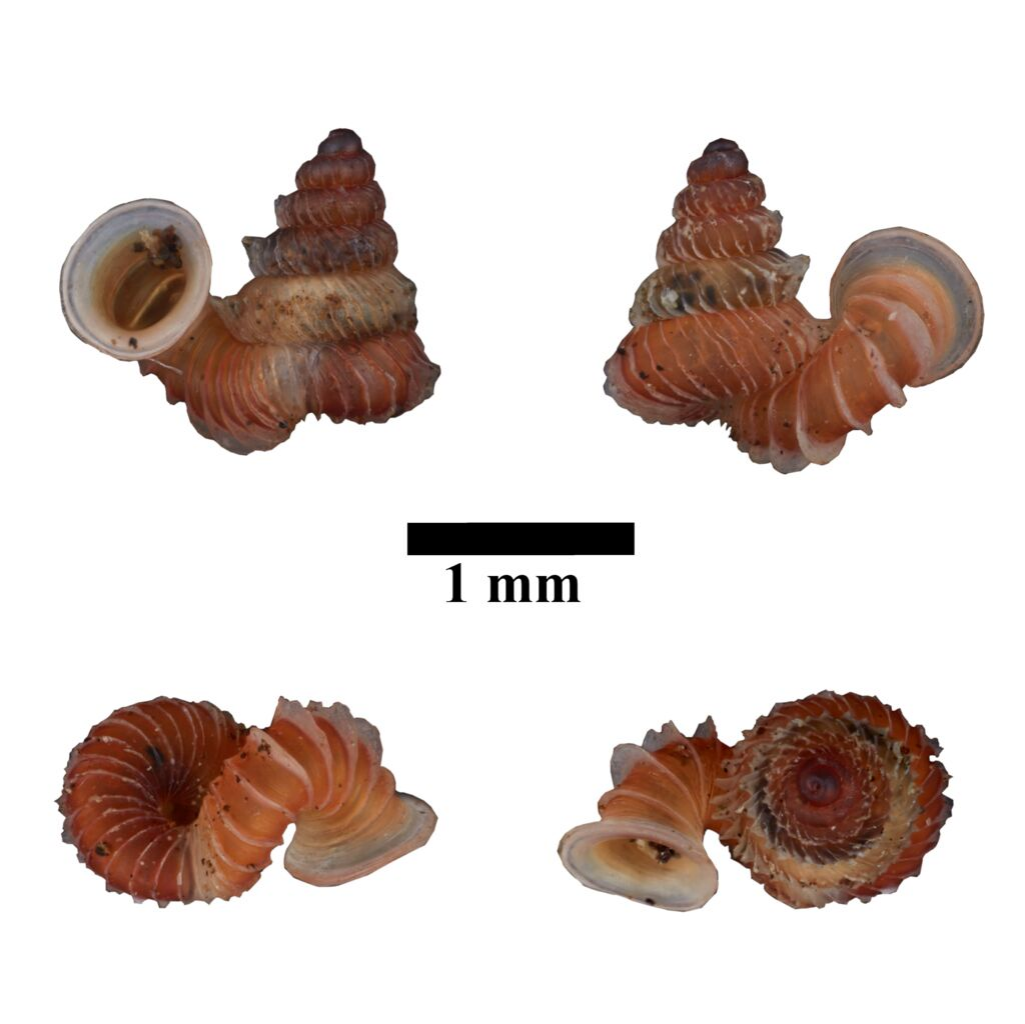
Apertural, posterial, umbilical and apical views of *Plectostomawallaceikudikense* subsp. nov. Holotype (MZU.MOL.21.17). Scale = 1 mm.

Living snails were observed on the wet limestone wall surfaces covered with mosses and lichens inside the collapsed cave. The representative is shown in Fig. 6.

## Checklists

### Checklists of land snails at Batu Kudik, Simunjan, Sarawak (Malaysia)

#### Acmella cyrtoglyphe

Vermeulen, Liew & Schilthuizen, 2015

C2EC4DAE-BA8A-5FC8-8380-E9B383DA1DA9

##### Materials

- **Type status:** Other material. **Occurrence:** catalogNumber: ME 13355; individualCount: 2; occurrenceID: 8872ABBA-9006-5389-A479-3AC539C478F6; **Taxon:** family: Assimineidae; genus: Acmella; scientificNameAuthorship: Vermeulen, Liew & Schilthuizen, 2015; **Location:** locality: Batu Kudik, Simunjan, Sarawak
- **Type status:** Other material. **Occurrence:** catalogNumber: ME 13890; individualCount: 1; occurrenceID: CF201FC2-21CD-59D1-91C1-39AD69E54E86; **Taxon:** family: Assimineidae; genus: Acmella; scientificNameAuthorship: Vermeulen, Liew & Schilthuizen, 2015; **Location:** locality: Batu Kudik, Simunjan, Sarawak

##### Distribution

Sarawak: Samarahan and Kuching Divisions (Vermeulen et al. 2015; Marzuki et al. 2021). Sabah: Interior, Sandakan and Tawau Divisions. **Distribution elsewhere**: Indonesia: Kalimantan (Vermeulen and Liew 2022). Endemic to Borneo.

##### Notes

**Figure 7.**
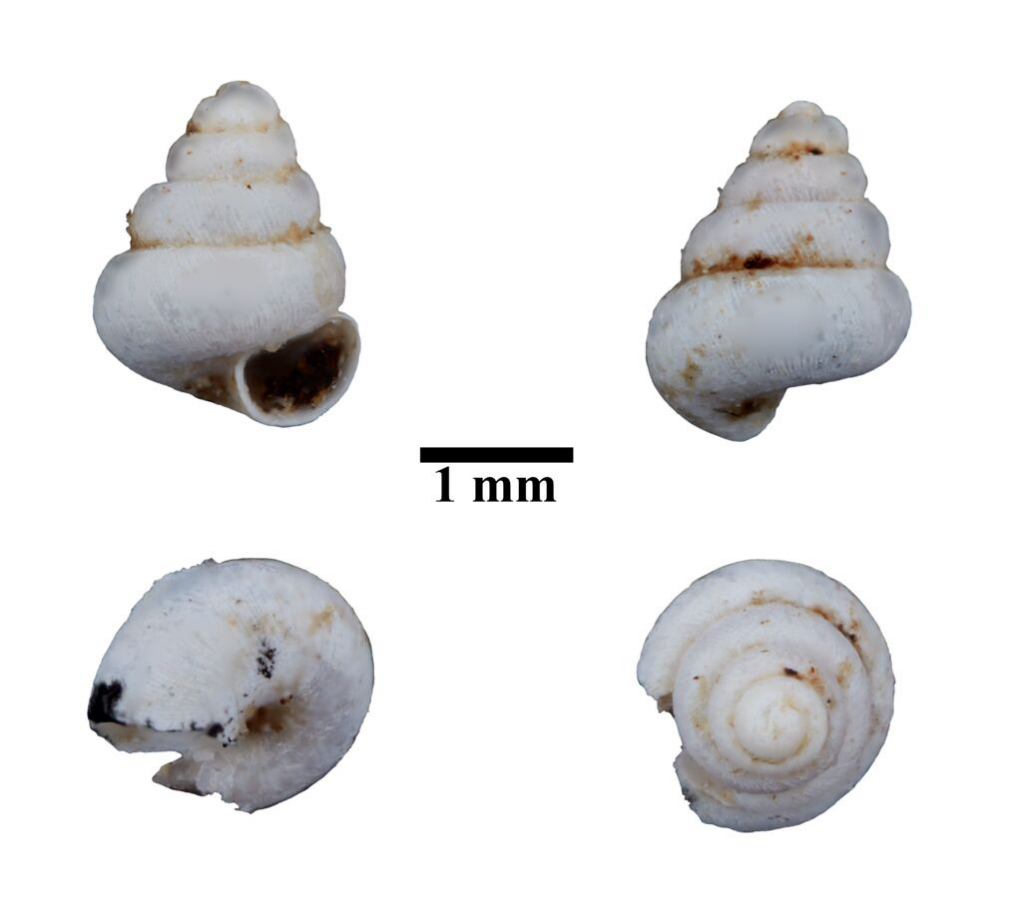
Apertural, posterial, umbilical and apical views of *Acmellacyrtoglyphe* (ME 13355). Scale = 1 mm.

Only dry shells were found during the surveys. The representative is shown in Fig. 7. The identification of the species was based on the original description by Vermeulen et al. (2015) and species description by Marzuki et al. (2021). Holotype, not seen (RMNH.5003948).

#### Japonia bellula

(E. von Martens, 1865)

3AF41515-D3E9-553E-A92A-BC6A9359D62B

##### Materials

- **Type status:** Other material. **Occurrence:** catalogNumber: MZU.MOL.22.12; individualCount: 5; occurrenceID: 1D92B0AD-0AC3-54E9-91F3-06828A34B5A2; **Taxon:** family: Cyclophoridae; genus: Japonia; scientificNameAuthorship: (E. von Martens, 1865); **Location:** locality: Batu Kudik, Simunjan, Sarawak
- **Type status:** Other material. **Occurrence:** catalogNumber: ME 13356; individualCount: 10; occurrenceID: 9046639D-277B-5C96-AC1C-6B218FFE63F6; **Taxon:** family: Cyclophoridae; genus: Japonia; scientificNameAuthorship: (E. von Martens, 1865); **Location:** locality: Batu Kudik, Simunjan, Sarawak
- **Type status:** Other material. **Occurrence:** catalogNumber: ME 13891; individualCount: 16; occurrenceID: 41018002-D7E2-5CC7-B77C-9A1B8336EDBF; **Taxon:** family: Cyclophoridae; genus: Japonia; scientificNameAuthorship: (E. von Martens, 1865); **Location:** locality: Batu Kudik, Simunjan, Sarawak

##### Distribution

Sarawak: Kuching Division. **Distribution elsewhere**: Indonesia: Kalimantan, Western Region (Martens 1865).

##### Notes

**Figure 8.**
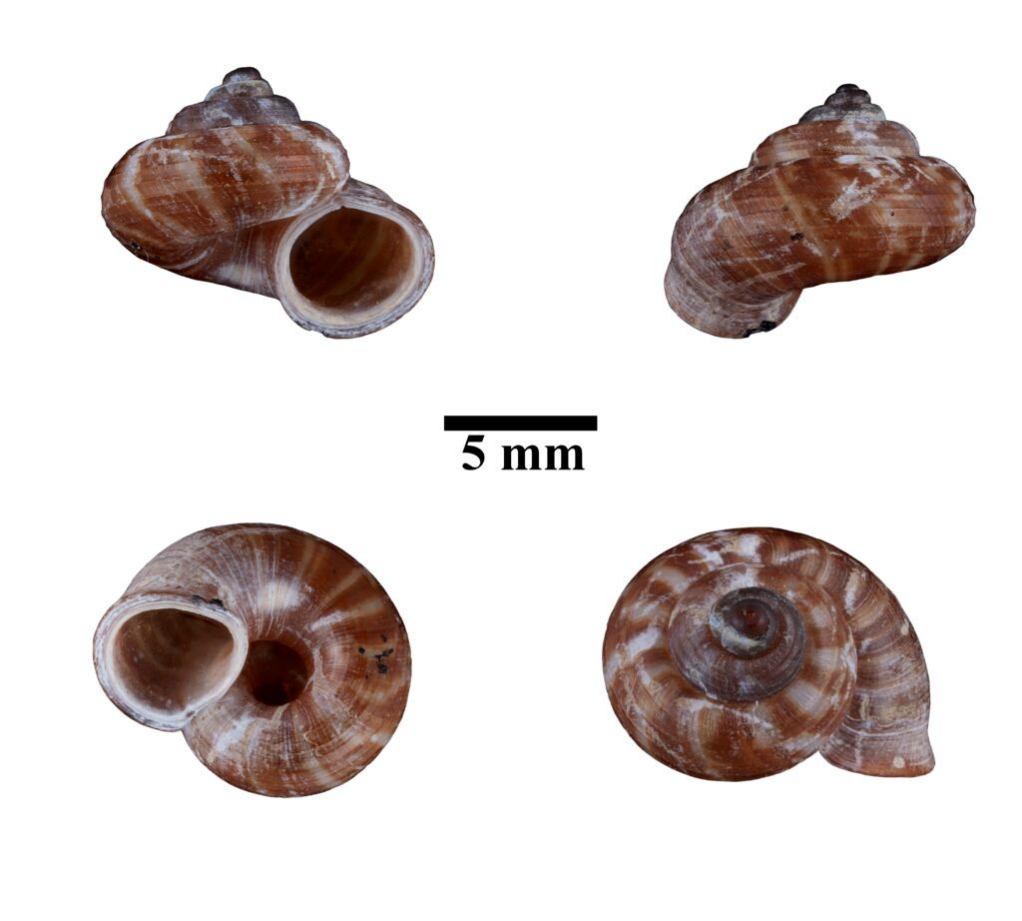
Apertural, posterial, umbilical and apical views of *Japoniabellula* (ME 13356). Scale = 5 mm.

Only dry shells were found during the surveys. The representative is shown in Fig. 8. The identification of the species was based on the original description by Martens (1865). Type specimen, not seen.

#### Stomacosmethis jagori

(E. von Martens, 1860)

B141D7C1-5D39-5411-87AD-330DA110B871

##### Materials

- **Type status:** Other material. **Occurrence:** catalogNumber: MZU.MOL.22.07; individualCount: 54; occurrenceID: CAEE2A19-A99C-5DCF-BFDF-D9A922AE23BB; **Taxon:** family: Alycaeidae; genus: Stomacosmethis; scientificNameAuthorship: (E. von Martens, 1860); **Location:** locality: Batu Kudik, Simunjan, Sarawak
- **Type status:** Other material. **Occurrence:** catalogNumber: ME 13357; individualCount: 68; occurrenceID: 06A0FCD4-9311-5503-A53C-72E50536895F; **Taxon:** family: Alycaeidae; genus: Stomacosmethis; scientificNameAuthorship: (E. von Martens, 1860); **Location:** locality: Batu Kudik, Simunjan, Sarawak
- **Type status:** Other material. **Occurrence:** catalogNumber: ME 13892; individualCount: 64; occurrenceID: D0F206B2-8A2F-5E1B-BDEB-5C7982BB61DF; **Taxon:** family: Alycaeidae; genus: Stomacosmethis; scientificNameAuthorship: (E. von Martens, 1860); **Location:** locality: Batu Kudik, Simunjan, Sarawak

##### Distribution

Widely distributed in Sarawak, Sabah: Sapulut and lower Kinabatangan. **Distribution elsewhere**: Indonesia: Kalimantan, Sumatra, Java and Bali (Martens 1860; Vermeulen and Liew 2022).

##### Notes

**Figure 9.**
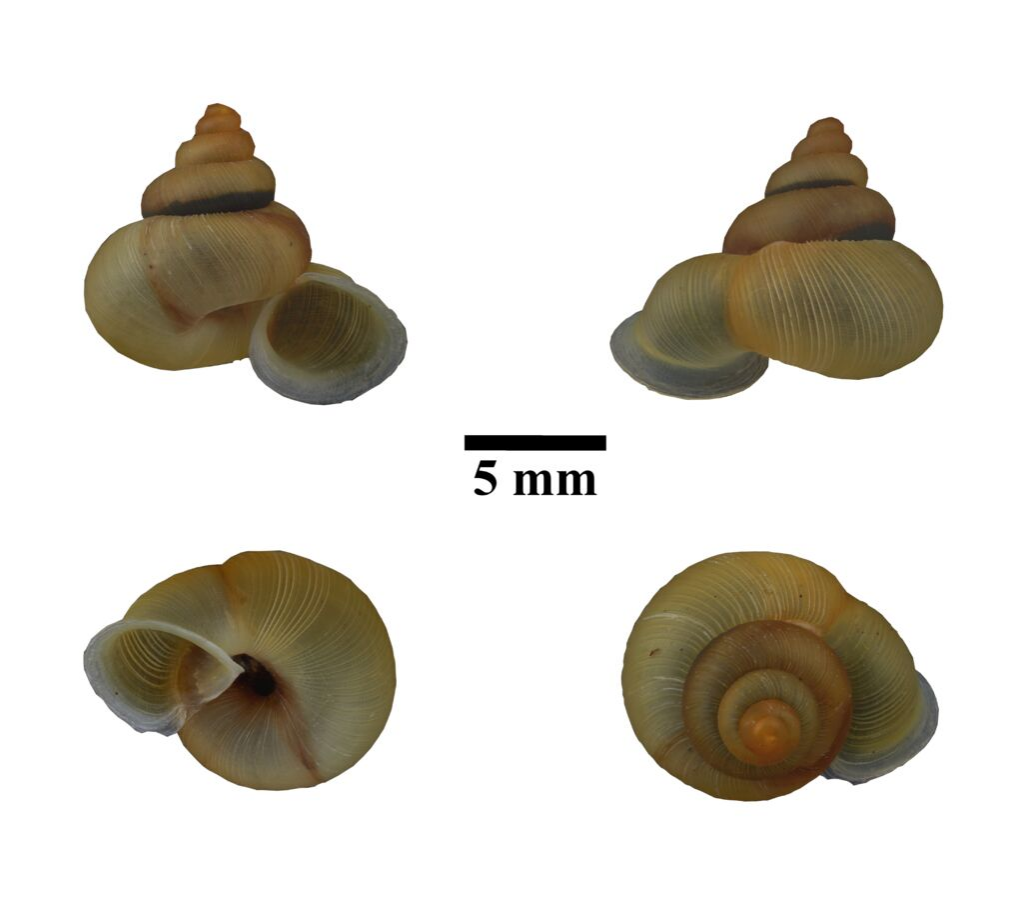
Apertural, posterial, umbilical and apical views of *Stomacosmethisjagori* (ME 13357). Scale = 5 mm.

Living snails were observed on the wet limestone wall surfaces covered with mosses and lichens. The representative is shown in Fig. 9. The identification of the species was based on the original description by Martens (1860). Type specimen, not seen.

#### Diplommatina concinna

H. Adams, 1872

52D8EF72-C840-5A10-934D-9B14D8061C58

##### Materials

- **Type status:** Other material. **Occurrence:** catalogNumber: MZU.MOL.22.06; individualCount: 150; occurrenceID: 3981DB7C-6B58-55A5-B6C0-1FE473EC2B32; **Taxon:** family: Diplommatinidae; genus: Diplommatina; scientificNameAuthorship: H. Adams, 1872; **Location:** locality: Batu Kudik, Simunjan, Sarawak
- **Type status:** Other material. **Occurrence:** catalogNumber: ME 13358; individualCount: 213; occurrenceID: 4D0570FC-6043-520A-8A36-E2194622B1B9; **Taxon:** family: Diplommatinidae; genus: Diplommatina; scientificNameAuthorship: H. Adams, 1872; **Location:** locality: Batu Kudik, Simunjan, Sarawak
- **Type status:** Other material. **Occurrence:** catalogNumber: ME 13893; individualCount: 189; occurrenceID: 15002878-9609-5177-A96F-D7701F8BC1AD; **Taxon:** family: Diplommatinidae; genus: Diplommatina; scientificNameAuthorship: H. Adams, 1872; **Location:** locality: Batu Kudik, Simunjan, Sarawak

##### Distribution

Sarawak: Kuching, Serian and Miri Divisions (Adams 1872; Marzuki et al. 2021). **Distribution elsewhere**: Indonesia: Bunguran Island.

##### Notes

**Figure 10.**
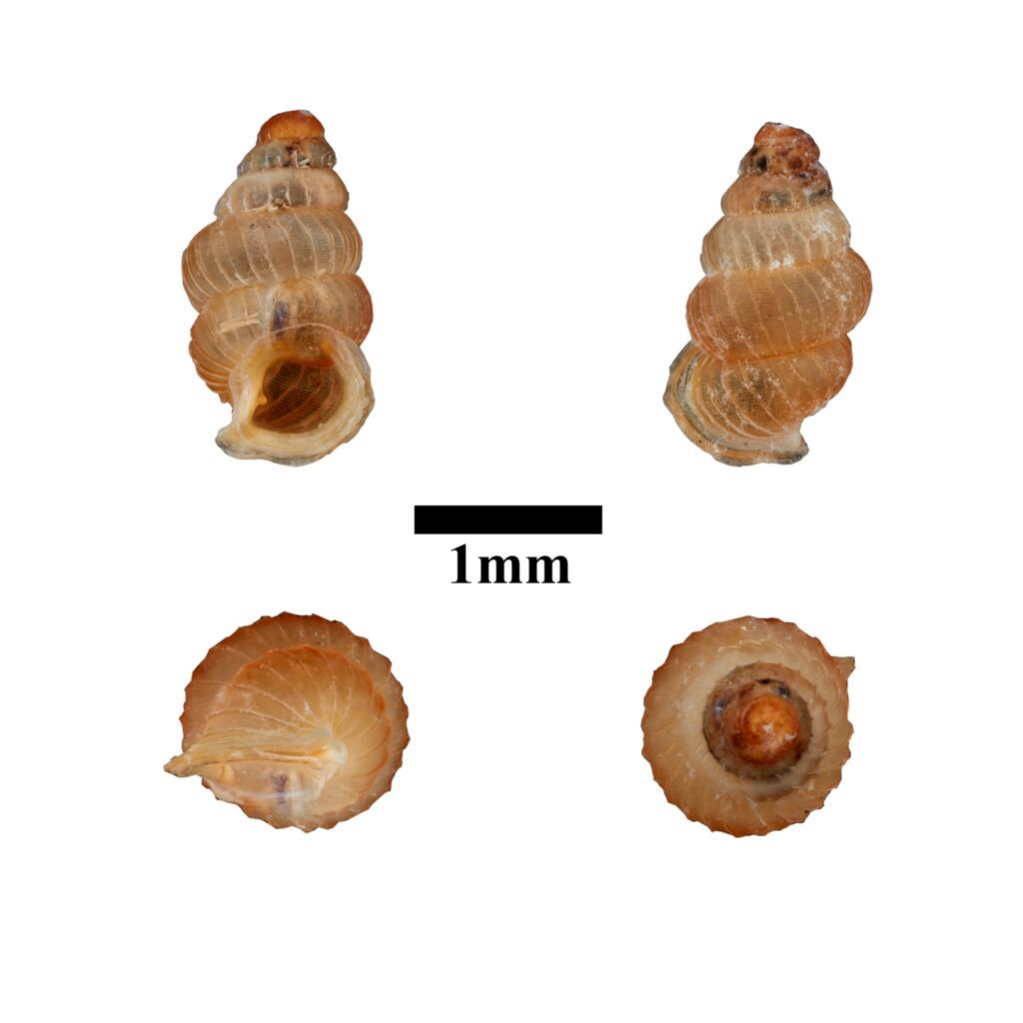
Apertural, posterial, umbilical and apical views of *Diplommatinaconcinna* (ME 13358). Scale = 1 mm.

Living snails were observed amongst the leaf litter and plant debris at the base of the limestone cliff. The representative is shown in Fig. 10. The identification of the species was based on the original description by Adams (1872), species description by Vermeulen (1993) and Marzuki et al. (2021). Holotype, not seen (BMNH 78.1.28.266.).

#### Diplommatina onyx

Fulton, 1901

AB991BBF-25A3-5B1C-B4DE-486D573BA9FC

##### Materials

- **Type status:** Other material. **Occurrence:** catalogNumber: MZU.MOL.22.193; individualCount: 1; occurrenceID: 1810A537-0BF5-5E71-848D-F06FCC9DE7E1; **Taxon:** family: Diplommatinidae; genus: Diplommatina; scientificNameAuthorship: Fulton, 1901; **Location:** locality: Batu Kudik, Simunjan, Sarawak

##### Distribution

Sarawak: Kuching, Serian, Samarahan and Miri Divisions (Fulton 1901; Vermeulen 1993; Marzuki et al. 2021). Endemic to Sarawak.

##### Notes

**Figure 11.**
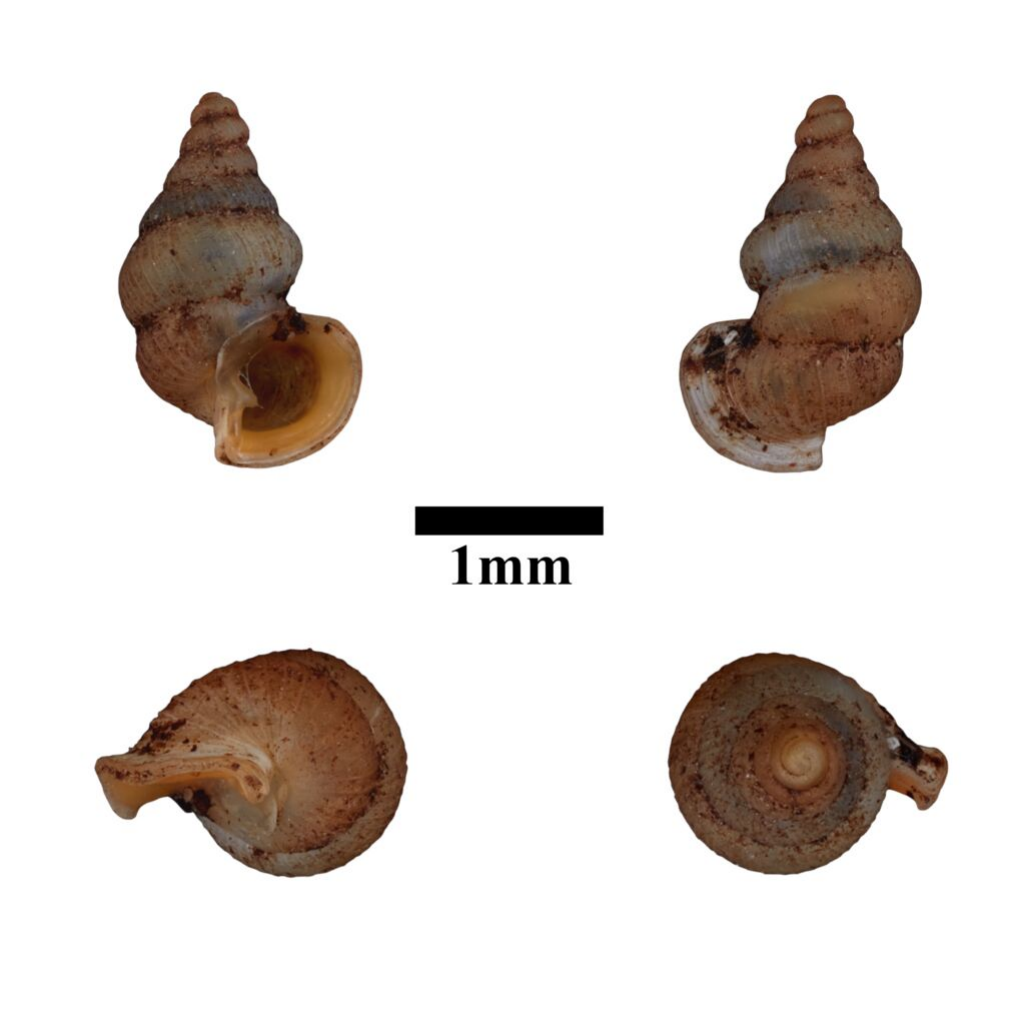
Apertural, posterial, umbilical and apical views of *Diplommatinaonyx* (MZU.MOL.22.193). Scale = 1 mm.

Only dry shells were found during the surveys. The representative is shown in Fig. 11. The identification of the species was based on the original description by Fulton (1901), species description by Vermeulen (1993) and Marzuki et al. (2021). Holotype, not seen (BMNH 1901.12.9.93.).

#### Opisthostoma javanica

Benthem-Jutting, 1932

3E408FD5-7548-5A4B-8103-678FED1B9307

##### Materials

- **Type status:** Other material. **Occurrence:** catalogNumber: MZU.MOL.22.192; individualCount: 1138; occurrenceID: DA3F927F-4ACA-5977-AAFD-7ACE5900A225; **Taxon:** family: Diplommatinidae; genus: Opisthostoma; scientificNameAuthorship: Benthem-Jutting, 1932; **Location:** locality: Batu Kudik, Simunjan, Sarawak
- **Type status:** Other material. **Occurrence:** catalogNumber: ME 13359; individualCount: 974; occurrenceID: 9D969EE8-F9F9-53E4-B938-59538C239EC9; **Taxon:** family: Diplommatinidae; genus: Opisthostoma; scientificNameAuthorship: Benthem-Jutting, 1932; **Location:** locality: Batu Kudik, Simunjan, Sarawak
- **Type status:** Other material. **Occurrence:** catalogNumber: ME13894; individualCount: 1410; occurrenceID: 9F31BD45-A97D-53F8-B5F4-F3818B50CAB7; **Taxon:** family: Diplommatinidae; genus: Opisthostoma; scientificNameAuthorship: Benthem-Jutting, 1932; **Location:** locality: Batu Kudik, Simunjan, Sarawak

##### Distribution

Sarawak: Samarahan Division. **Distribution elsewhere**: Indonesia: Kalimantan, Java, Madura and Celebes (Vermeulen 1991).

##### Notes

**Figure 12.**
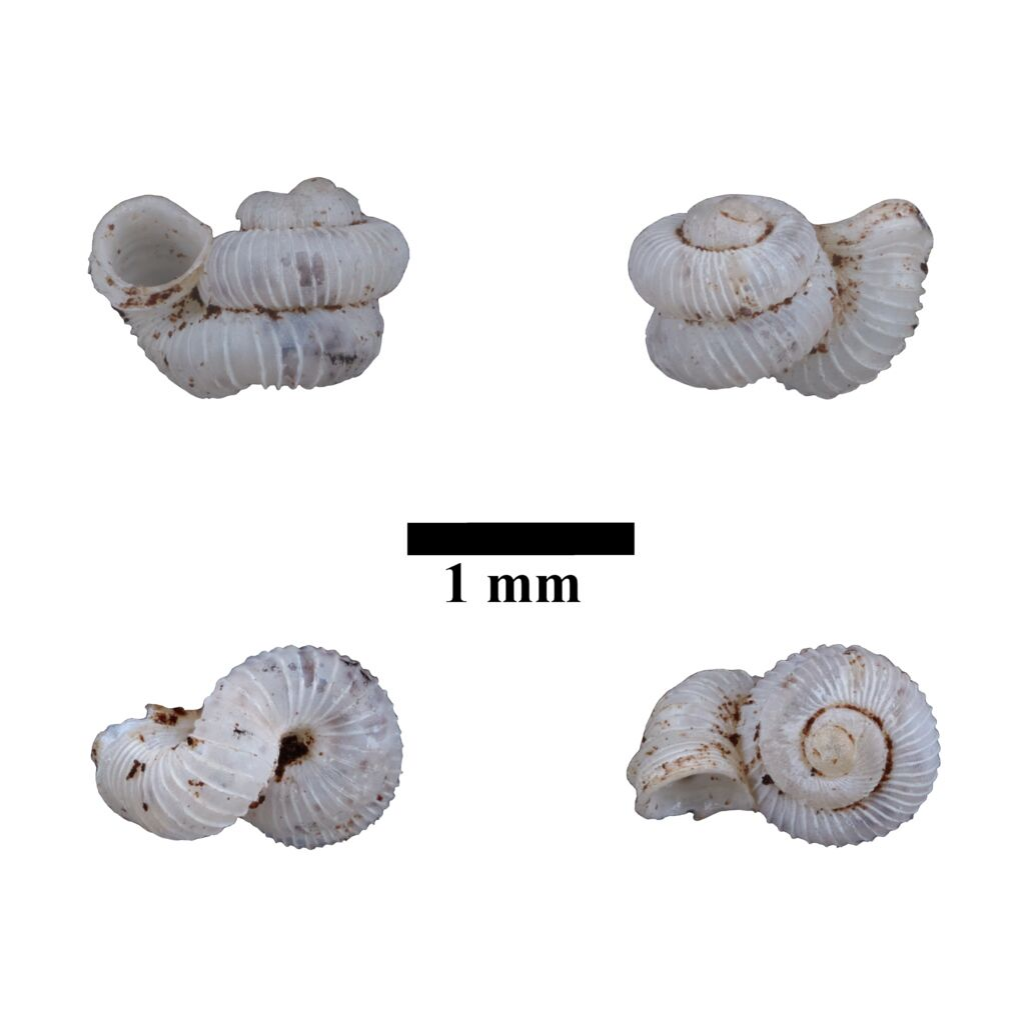
Apertural, posterial, umbilical and apical views of *Opisthostomajavanica* (MZU.MOL.22.192). Scale = 1 mm.

Living snails were observed amongst the leaf litter and plant debris inside the collapsed cave. Bornean *Opisthostomajavanica* Benthem-Jutting, 1932 has constriction with transverse palatalis together with infracolumellaris. Nurinsiyah and Hausdorf (2017) mentioned that the infracolumellaris was not observed in the Javan population. However, the shell radial ribs are widely spaced (4-6 ribs/0.5 mm on the penultimate whorl) and with distinctly elevated top whorls compared to Javan *Opisthostomajavanica*. Some specimens characterised by the rather upward-turned aperture similar to some *Opisthostoma* occurring in Peninsular Malaysia. The representative is shown in Fig. 12. The identification of the species was based on the original description of the species by van Benthem-Jutting (1932) and species description by Vermeulen (1991). Syntype, not seen (ZMA 136008).

#### Georissa hungerfordi

Godwin-Austen, 1889

FAE61032-B357-5C05-8E8B-66EFF603F8F7

##### Materials

- **Type status:** Other material. **Occurrence:** catalogNumber: MZU.MOL.22.11; individualCount: 2; occurrenceID: B902587F-2AB8-5AD8-822F-2EF73710611E; **Taxon:** family: Hydrocenidae; genus: Georissa; scientificNameAuthorship: Godwin-Austen, 1889; **Location:** locality: Batu Kudik, Simunjan, Sarawak
- **Type status:** Other material. **Occurrence:** catalogNumber: ME 13353; individualCount: 9; occurrenceID: 9601FC42-39C3-56EA-BB3C-B906CE915A07; **Taxon:** family: Hydrocenidae; genus: Georissa; scientificNameAuthorship: Godwin-Austen, 1889; **Location:** locality: Batu Kudik, Simunjan, Sarawak
- **Type status:** Other material. **Occurrence:** catalogNumber: ME 13888; individualCount: 33; occurrenceID: 0D0459EB-BA81-5DEF-B5E4-052F21C30F73; **Taxon:** family: Hydrocenidae; genus: Georissa; scientificNameAuthorship: Godwin-Austen, 1889; **Location:** locality: Batu Kudik, Simunjan, Sarawak

##### Distribution

Sarawak: Kuching, Serian and Samarahan Divisions (Godwin-Austen 1889; Khalik et al. 2019; Marzuki et al. 2021). Endemic to western Sarawak.

##### Notes

**Figure 13.**
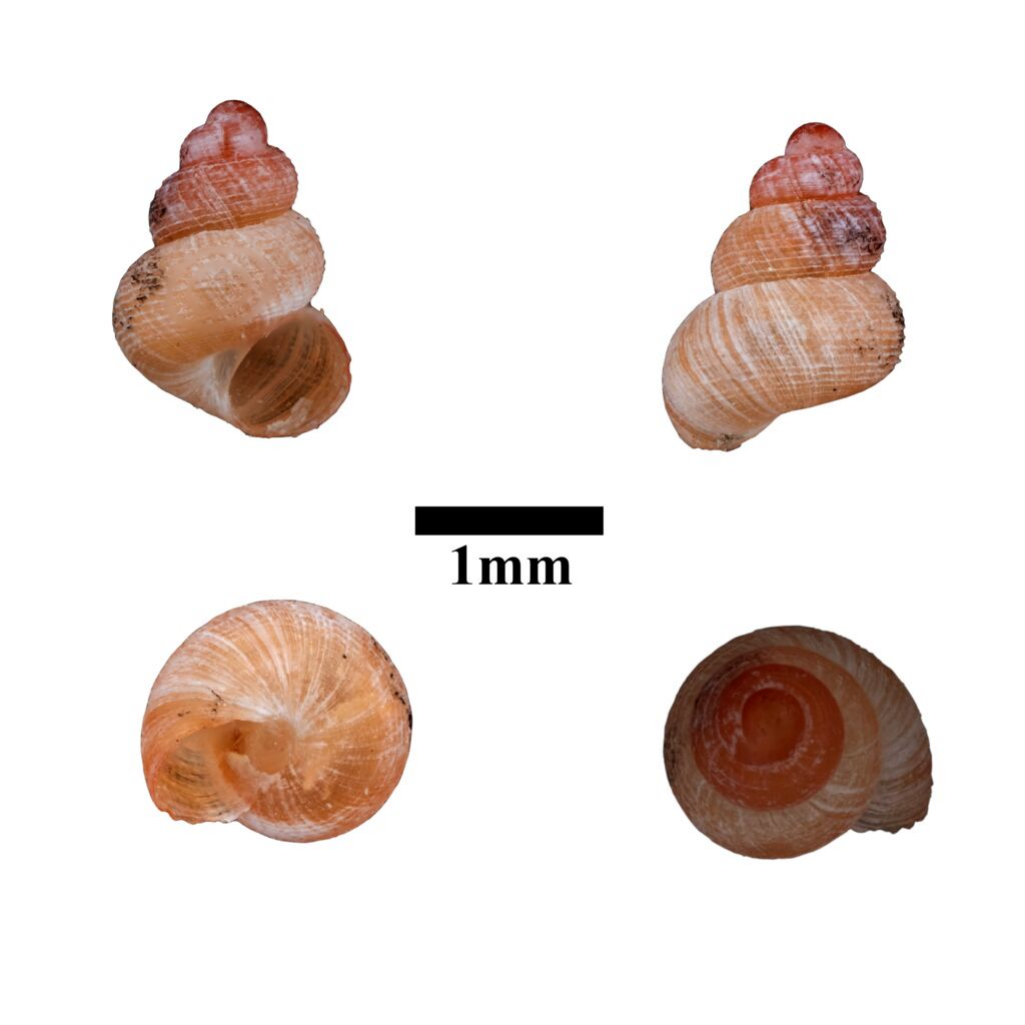
Apertural, posterial, umbilical and apical views of *Georissahungerfordi* (ME 13353). Scale = 1 mm.

Only dry shells were found during the surveys. The representative is shown in Fig. 13. The identification of the species was based on the original description by Godwin-Austen (1889), species description by Khalik et al. (2019) and Marzuki et al. (2021). Lectotype, seen (NHMUK 1891.3.17.864).

#### Georissa pyrrhoderma

Thompson & Dance, 1983

1B5C48DF-6756-5EC3-8EFD-551791A85F8E

##### Materials

- **Type status:** Other material. **Occurrence:** catalogNumber: MZU.MOL.22.08; individualCount: 987; occurrenceID: 699683AF-EE08-5CAD-8523-63D16C81639C; **Taxon:** family: Hydrocenidae; genus: Georissa; scientificNameAuthorship: Thompson & Dance, 1983; **Location:** locality: Batu Kudik, Simunjan, Sarawak
- **Type status:** Other material. **Occurrence:** catalogNumber: ME 13354; individualCount: 1050; occurrenceID: 597B12A2-1DFC-50A8-A418-63EDFAA91D95; **Taxon:** family: Hydrocenidae; genus: Georissa; scientificNameAuthorship: Thompson & Dance, 1983; **Location:** locality: Batu Kudik, Simunjan, Sarawak
- **Type status:** Other material. **Occurrence:** catalogNumber: ME 13889; individualCount: 1033; occurrenceID: 70EA2F8C-D1B0-52A3-8596-7158FFE37797; **Taxon:** family: Hydrocenidae; genus: Georissa; scientificNameAuthorship: Thompson & Dance, 1983; **Location:** locality: Batu Kudik, Simunjan, Sarawak

##### Distribution

Sarawak: Serian and Samarahan Divisions (Thompson and Dance 1983; Khalik et al. 2018). Endemic to western Sarawak.

##### Notes

**Figure 14.**
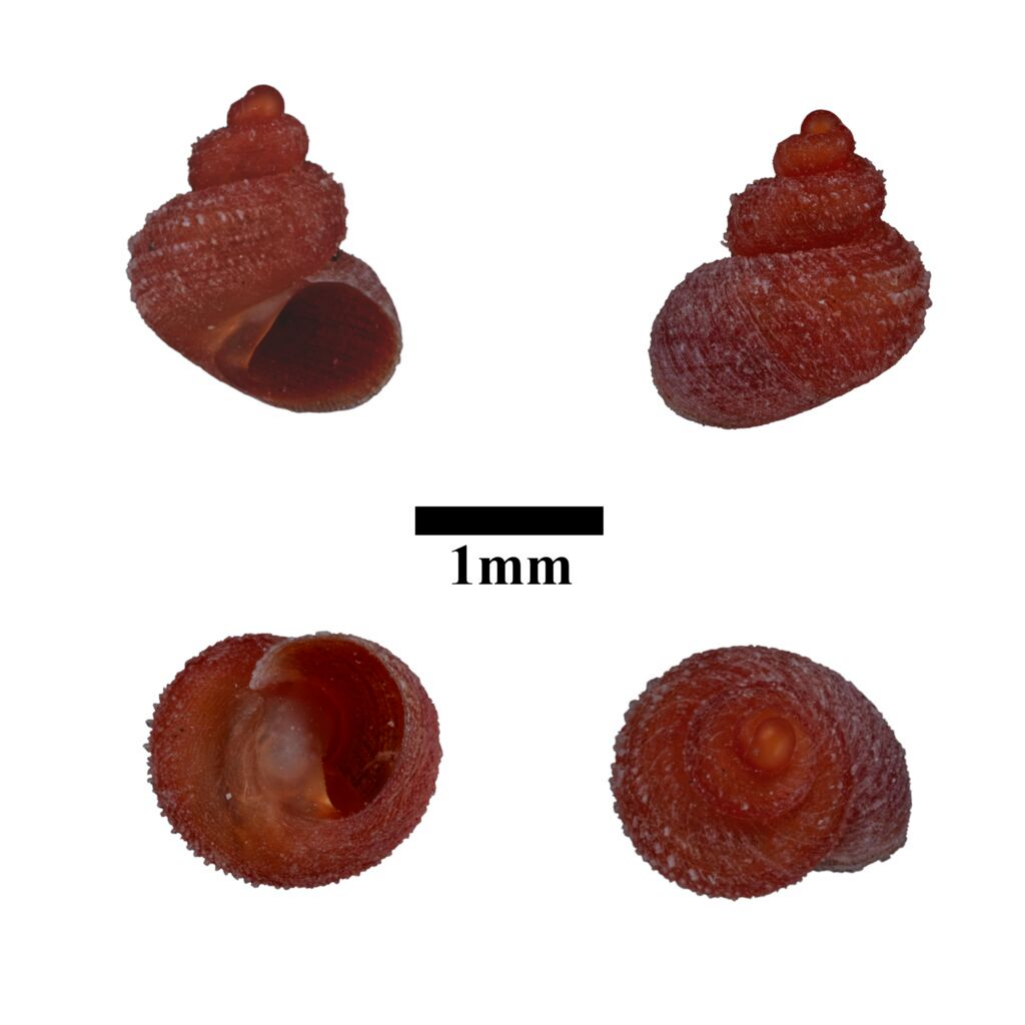
Apertural, posterial, umbilical and apical views of *Georissapyrrhoderma* (ME 13354). Scale = 1 mm.

Living snails were observed on the wet limestone wall surfaces covered with mosses and lichens. This marked the second locality record of this species after the type locality. The representative is shown in Fig. 14. The identification of the species was based on the original description by Thompson and Dance (1983), species description by Beron (2015) and Khalik et al. (2018). Paratype, not seen (NHMUK1984005).

#### Allopeas gracile

(Hutton, 1834)

4E33EEBA-203D-57E7-B42C-A25DFB41173A

##### Materials

- **Type status:** Other material. **Occurrence:** catalogNumber: MZU.MOL.22.09; individualCount: 1; occurrenceID: 19B635C9-85D4-5EC7-B2C5-474C84D48304; **Taxon:** family: Achatinidae; genus: Allopeas; scientificNameAuthorship: (Hutton, 1834); **Location:** locality: Batu Kudik, Simunjan, Sarawak
- **Type status:** Other material. **Occurrence:** catalogNumber: ME 13365; individualCount: 20; occurrenceID: 7D699A5D-067F-5650-A3C0-DB24A587E7CE; **Taxon:** family: Achatinidae; genus: Allopeas; scientificNameAuthorship: (Hutton, 1834); **Location:** locality: Batu Kudik, Simunjan, Sarawak
- **Type status:** Other material. **Occurrence:** catalogNumber: ME 13900; individualCount: 25; occurrenceID: 12B5C88E-AD84-586A-95F2-3AF5C8EBCF05; **Taxon:** family: Achatinidae; genus: Allopeas; scientificNameAuthorship: (Hutton, 1834); **Location:** locality: Batu Kudik, Simunjan, Sarawak

##### Distribution

Sarawak: Kuching, Serian, Samarahan, Mukah and Miri Divisions (Marzuki et al. 2021). Sabah: Interior, Kudat, Sandakan, Tawau and West Coast Divisions (Vermeulen and Liew 2022). **Distribution elsewhere**: Indonesia: West Kalimantan Provinces and circumtropical (Hutton 1834; Vermeulen and Whitten 1998; Vermeulen and Liew 2022).

##### Notes

**Figure 15.**
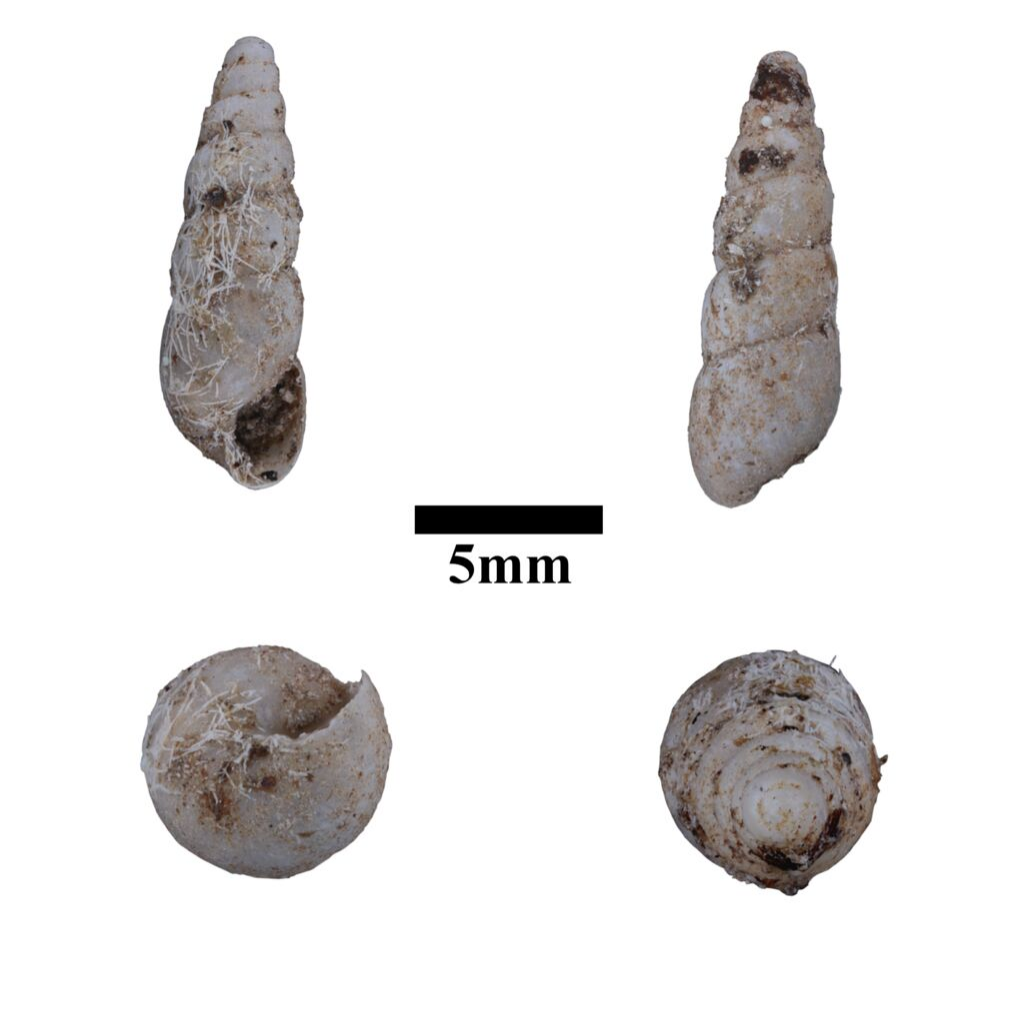
Apertural, posterial, umbilical and apical views of *Allopeasgracile* (MZU.MOL.22.09). Scale = 5 mm.

Widespread through­out Borneo. Living snails were observed amongst the leaf litter and plant debris near the cliff in a lowland limestone forest. The representative is shown in Fig. 15. The identification of the species was based on the original description by Hutton (1834) and species description by Marzuki et al. (2021). Syntype, not seen (NHMUK1856.9.15.68).

#### Allopeas clavulinum

(Potiez & Michaud, 1838)

2251D8E2-E00D-5F29-9913-C14F13CC99F8

##### Materials

- **Type status:** Other material. **Occurrence:** catalogNumber: MZU.MOL.22.10; individualCount: 3; occurrenceID: FB3CED1F-8D21-5413-A53E-0C3C6C5AEA6D; **Taxon:** family: Achatinidae; genus: Allopeas; scientificNameAuthorship: (Potiez & Michaud, 1838); **Location:** locality: Batu Kudik, Simunjan, Sarawak
- **Type status:** Other material. **Occurrence:** catalogNumber: ME 13366; individualCount: 23; occurrenceID: D39E9F60-49E2-5C84-87C9-F3FECD06316A; **Taxon:** family: Achatinidae; genus: Allopeas; scientificNameAuthorship: (Potiez & Michaud, 1838); **Location:** locality: Batu Kudik, Simunjan, Sarawak
- **Type status:** Other material. **Occurrence:** catalogNumber: ME 13901; individualCount: 5; occurrenceID: 70E85BA3-FF59-52E4-8085-DBF9A21F6691; **Taxon:** family: Achatinidae; genus: Allopeas; scientificNameAuthorship: (Potiez & Michaud, 1838); **Location:** locality: Batu Kudik, Simunjan, Sarawak

##### Distribution

Sarawak: Kuching, Serian, Samarahan, Sibu and Miri Divisions (Marzuki et al. 2021). Sabah: Interior, Sandakan, Tawau, Kudat and West Coast Divisions (Vermeulen and Liew 2022). **Distribution elsewhere**: Africa, Asia, Australia and Pacific Islands. (Potiez and Michaud 1838; Vermeulen and Whitten 1998; Vermeulen and Liew 2022).

##### Notes

**Figure 16.**
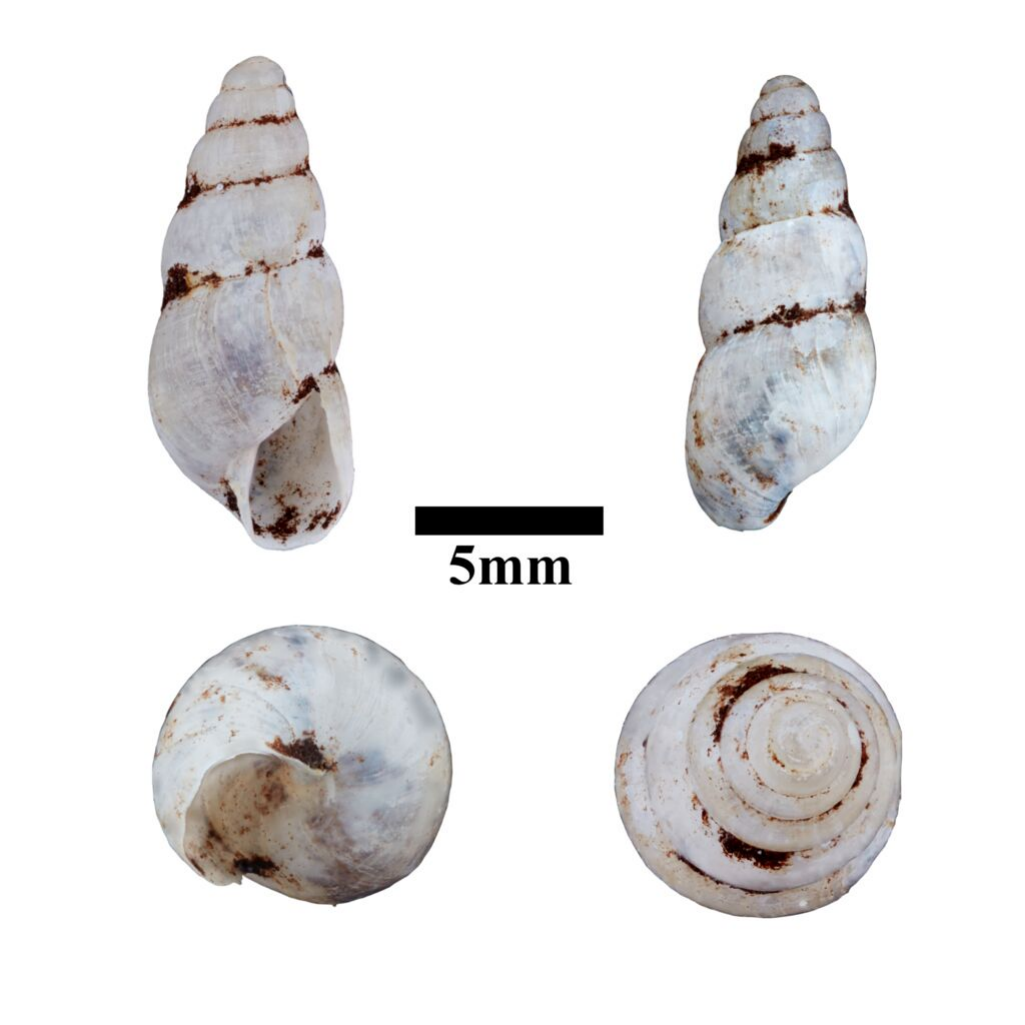
Apertural, posterial, umbilical and apical views of *Allopeasclavulinum* (MZU.MOL.22.10). Scale = 5 mm.

An introduced species. Widespread through­out Borneo. Only dry shells were found during the surveys. The representative is shown in Fig. 16. The identification of the species was based on the original description by Potiez and Michaud (1838) and species description by Marzuki et al. (2021). Type specimen, not seen.

#### Hemiplecta densa

(H. Adams & Reeve, 1850)

202A572D-87BE-5540-814B-A48FB053B181

##### Materials

- **Type status:** Other material. **Occurrence:** catalogNumber: MZU.MOL.22.18; individualCount: 2; occurrenceID: E8D5B1BE-6672-5B4F-88B5-E844B6E70AD9; **Taxon:** family: Ariophantidae; genus: Hemiplecta; scientificNameAuthorship: (H. Adams & Reeve, 1850); **Location:** locality: Batu Kudik, Simunjan, Sarawak

##### Distribution

Sarawak: Kuching, Serian and Samarahan Divisions (Marzuki et al. 2021). Sabah: West Coast, Kudat, Interior, Sandakan and Tawau Divisions (Vermeulen and Liew 2022). **Distribution elsewhere**: Indonesia: West and East Kalimantan Provinces, Java, Sumatra and the Philippines (Adams and Reeve 1850; Mousson 1857; Smith 1895).

##### Notes

**Figure 17.**
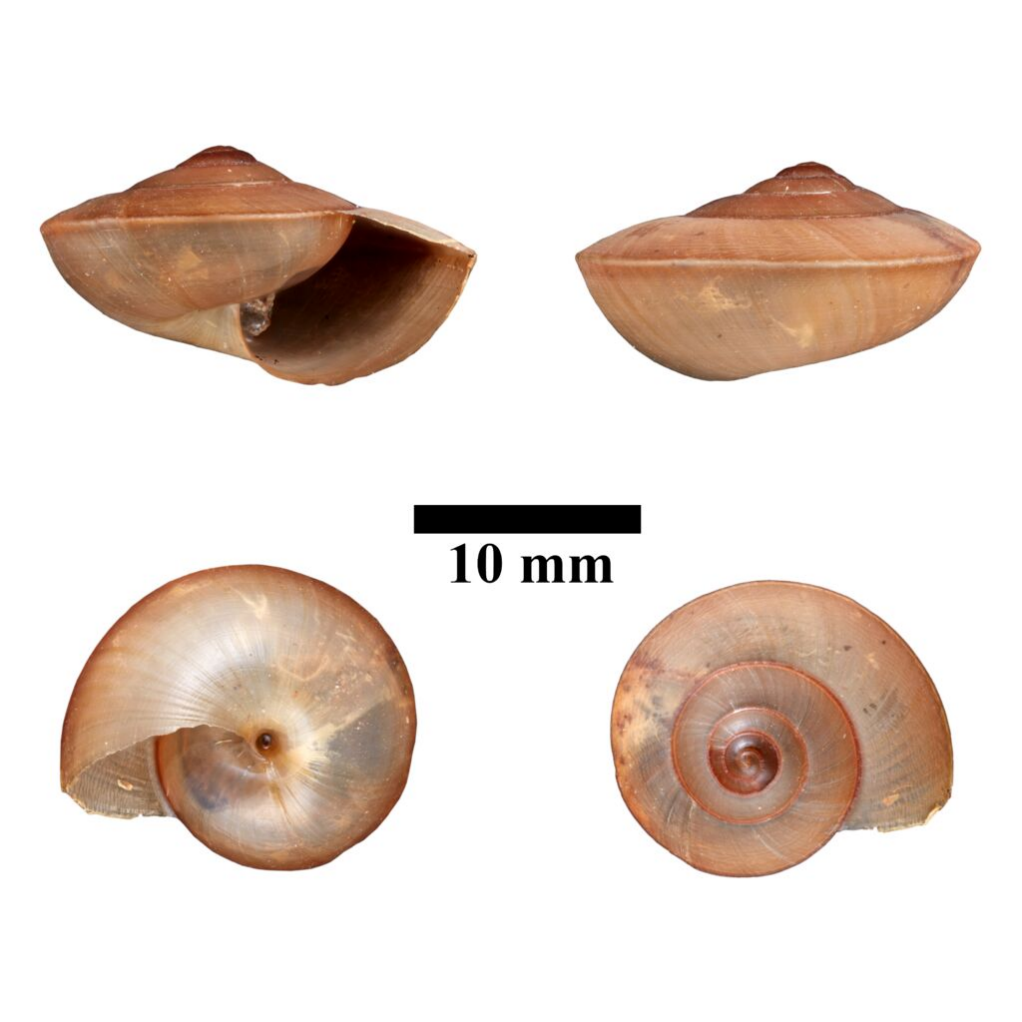
Apertural, posterial, umbilical and apical views of *Hemiplectadensa* (MZU.MOL.22.18). Scale = 10 mm.

Only dry shells were found during the surveys. The representative is shown in Fig. 17. The identification of the species was based on the original description by Adams and Reeve (1850) and species description by Marzuki et al. (2021). Type specimen, not seen.

#### Macrochlamys infans

(Reeve, 1854)

98E3A1E2-0C3E-5BCD-9368-6C4162644F69

##### Materials

- **Type status:** Other material. **Occurrence:** catalogNumber: MZU.MOL.22.14; individualCount: 2; occurrenceID: 408EDBCE-1495-53F5-83B9-9571EC1C705F; **Taxon:** family: Ariophantidae; genus: Macrochlamys; scientificNameAuthorship: (Reeve, 1854); **Location:** locality: Batu Kudik, Simunjan, Sarawak
- **Type status:** Other material. **Occurrence:** catalogNumber: ME 13363; individualCount: 3; occurrenceID: 04B7E3D0-0014-5318-BB95-B82655CD9927; **Taxon:** family: Ariophantidae; genus: Macrochlamys; scientificNameAuthorship: (Reeve, 1854); **Location:** locality: Batu Kudik, Simunjan, Sarawak
- **Type status:** Other material. **Occurrence:** catalogNumber: ME 13898; individualCount: 3; occurrenceID: B94B3B74-B564-541F-BB8A-E05761432E5B; **Taxon:** family: Ariophantidae; genus: Macrochlamys; scientificNameAuthorship: (Reeve, 1854); **Location:** locality: Batu Kudik, Simunjan, Sarawak

##### Distribution

Sarawak: Kuching, Serian, Samarahan and Miri Divisions (Reeve 1854; Marzuki et al. 2021). Sabah: Kudat, West Coast, Interior and Tawau Divisions (Vermeulen and Liew 2022). Endemic to Borneo.

##### Notes

**Figure 18.**
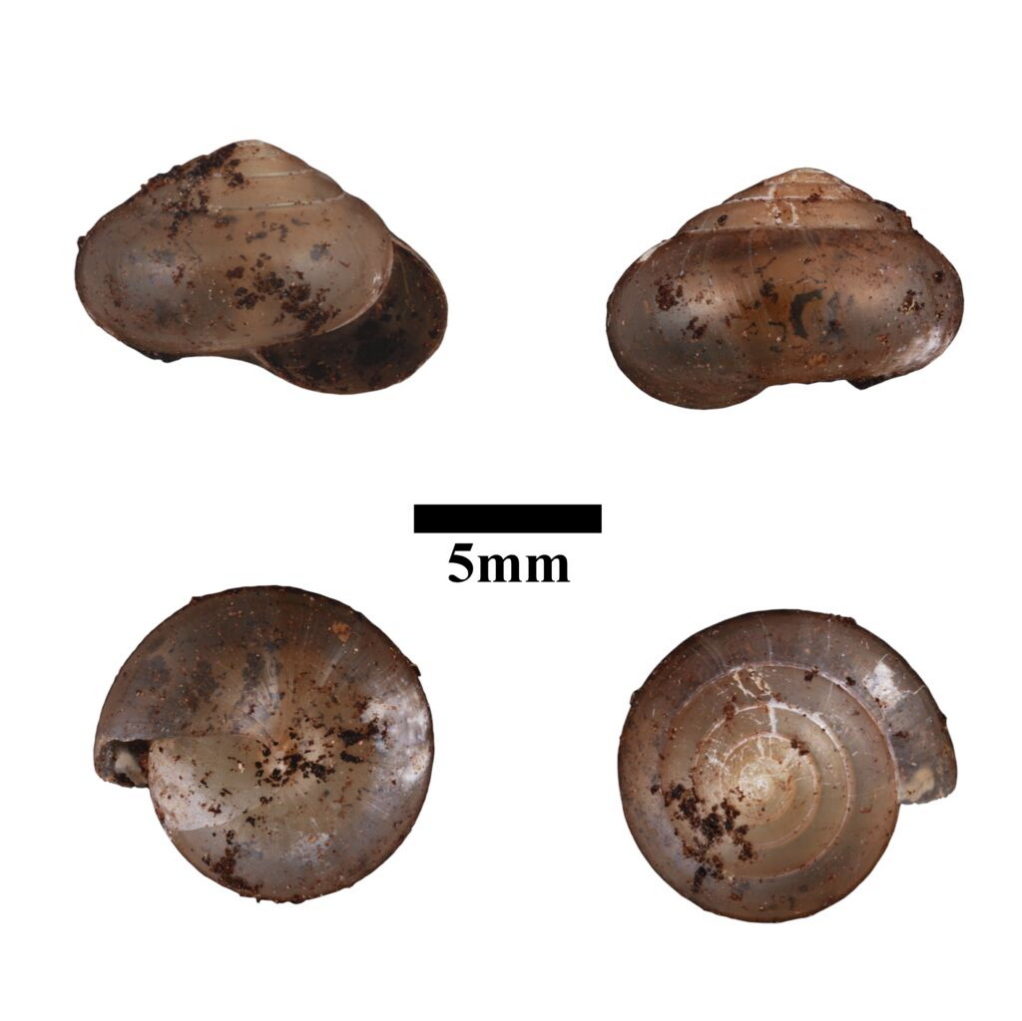
Apertural, posterial, umbilical and apical views of *Macrochlamysinfans* (ME 13363). Scale = 5 mm.

Living snails were observed amongst the leaf-litter and plant debris at the base of the limestone hill cliff. The representative is shown in Fig. 18. The identification of the species was based on the original description by Reeve (1854) and species descriptions by Marzuki et al. (2021). Type specimen, not seen.

#### Microcystina paripari

Marzuki, Liew & Mohd-Azlan, 2021

152FD73B-BC22-5A4E-81F6-633907038B66

##### Materials

- **Type status:** Other material. **Occurrence:** catalogNumber: MZU.MOL.22.06; individualCount: 2; occurrenceID: 2347090F-E3CA-585B-A8BB-666A1981831B; **Taxon:** family: Ariophantidae; genus: Microcystina; scientificNameAuthorship: Marzuki, Liew & Mohd-Azlan, 2021; **Location:** locality: Batu Kudik, Simunjan, Sarawak
- **Type status:** Other material. **Occurrence:** catalogNumber: ME 13364; individualCount: 5; occurrenceID: A277D6B6-649E-599F-BEAC-657B09E1E0A7; **Taxon:** family: Ariophantidae; genus: Microcystina; scientificNameAuthorship: Marzuki, Liew & Mohd-Azlan, 2021; **Location:** locality: Batu Kudik, Simunjan, Sarawak

##### Distribution

Sarawak: Kuching and Samarahan Divisions (Marzuki et al. 2021). Endemic to western Sarawak.

##### Notes

**Figure 19.**
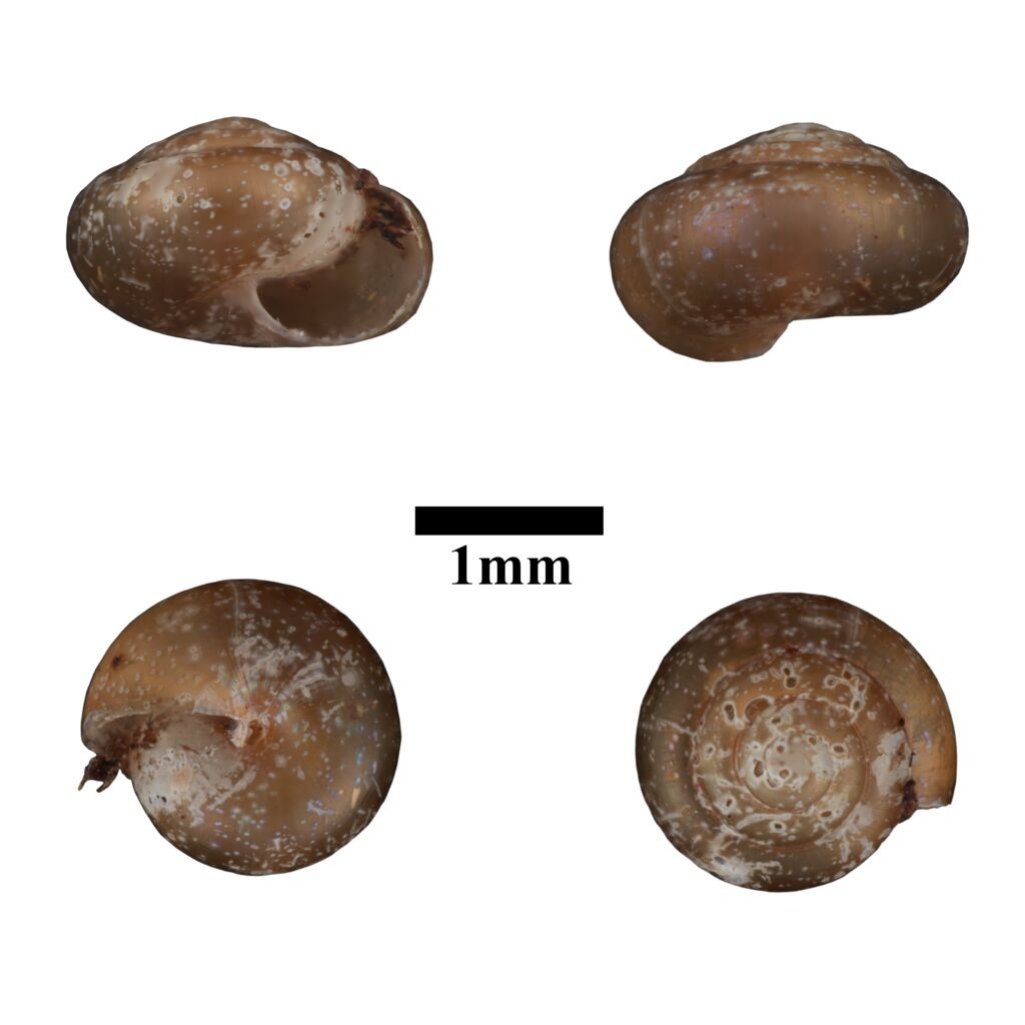
Apertural, posterial, umbilical and apical views of *Microcystinaparipari* (ME 13364). Scale = 1 mm.

Only dry shells were found during the surveys. The representative is shown in Fig. 19. The identification of the species was based on the original description by Marzuki et al. (2021). Holotype, seen (MZU.MOL.20.12).

#### Helicarion dyakanum

(Godwin-Austen, 1891)

21C1FBCE-A8B6-52A6-A7D4-2869B54D2625

##### Materials

- **Type status:** Other material. **Occurrence:** catalogNumber: MZU.MOL.22.452; individualCount: 1; occurrenceID: 9501608E-3784-54A9-8992-A699C780D0B6; **Taxon:** family: Helicarionidae; genus: Helicarion; scientificNameAuthorship: (Godwin-Austen, 1891); **Location:** locality: Batu Kudik, Simunjan, Sarawak
- **Type status:** Other material. **Occurrence:** catalogNumber: ME 13367; individualCount: 5; occurrenceID: FF103876-92B6-5BC6-B205-3DFBC37D5D70; **Taxon:** family: Helicarionidae; genus: Helicarion; scientificNameAuthorship: (Godwin-Austen, 1891); **Location:** locality: Batu Kudik, Simunjan, Sarawak
- **Type status:** Other material. **Occurrence:** catalogNumber: ME 13899; individualCount: 2; occurrenceID: 8E622615-3086-5C96-B118-8EC3F0636AD5; **Taxon:** family: Helicarionidae; genus: Helicarion; scientificNameAuthorship: (Godwin-Austen, 1891); **Location:** locality: Batu Kudik, Simunjan, Sarawak

##### Distribution

Sarawak: Kuching, Serian, Samarahan and Miri Divisions (Godwin-Austen 1891; Marzuki et al. 2021). Sabah: West Coast Division (Vermeulen and Liew 2022). **Distribution elsewhere**: Indonesia: Lombok (Smith 1899).

##### Notes

**Figure 20.**
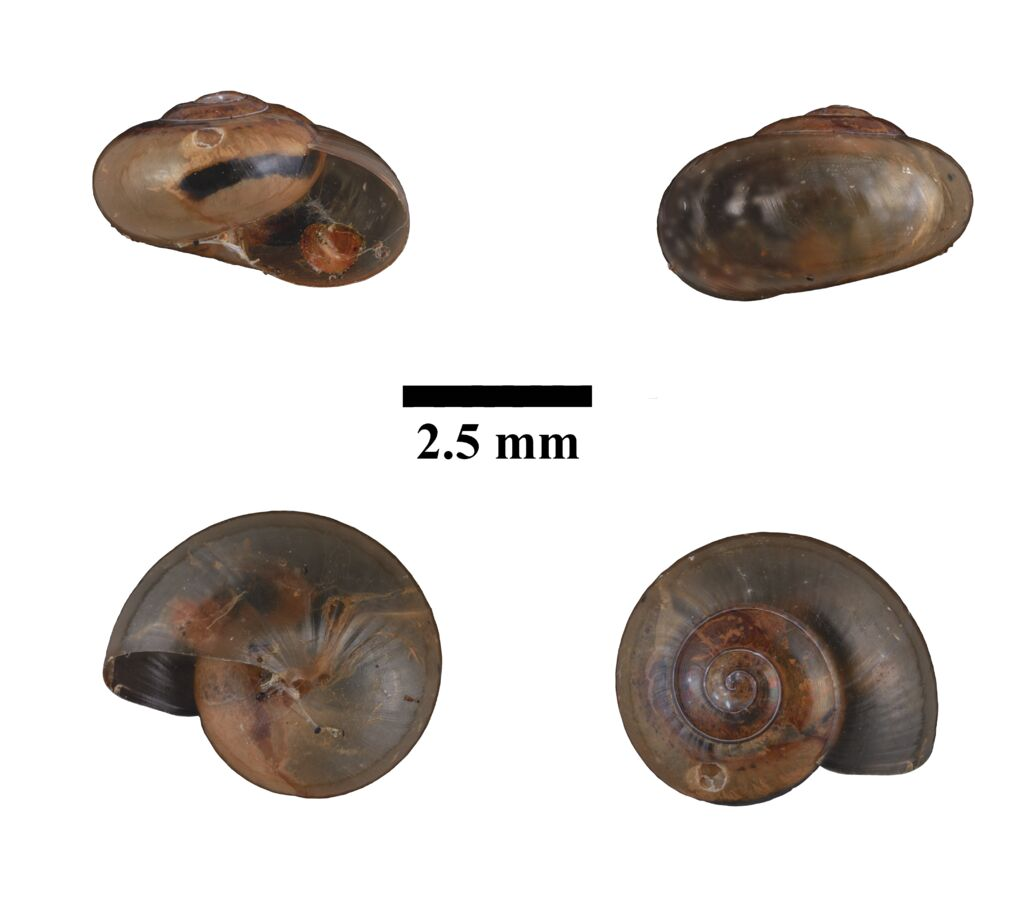
Apertural, posterial, umbilical and apical views of *Helicariondyakanum* (ME 13367). Scale = 2.5 mm.

Living snails were observed within the arboreal area, on leaves of palms or trees at the base of the limestone cliff. The representative is shown in Fig. 20. The identification of the species was based on the original description by Godwin-Austen (1891) and species description by Marzuki et al. (2021). Holotype, not seen (NHMUK 91.3.9.4).

#### Landouria winteriana

(Pfeiffer, 1842)

8125E478-2F88-5D32-AB15-522DAE024606

##### Materials

- **Type status:** Other material. **Occurrence:** catalogNumber: MZU.MOL.22.13; individualCount: 6; occurrenceID: DD783247-DF63-5D9E-B49E-DFF82F571952; **Taxon:** family: Camaenidae; genus: Landouria; scientificNameAuthorship: (Pfeiffer, 1842); **Location:** locality: Batu Kudik, Simunjan, Sarawak
- **Type status:** Other material. **Occurrence:** catalogNumber: ME 13369; individualCount: 1; occurrenceID: 4C193EE9-FDD8-5575-91C0-373E4F5E14C9; **Taxon:** family: Camaenidae; genus: Landouria; scientificNameAuthorship: (Pfeiffer, 1842); **Location:** locality: Batu Kudik, Simunjan, Sarawak
- **Type status:** Other material. **Occurrence:** catalogNumber: ME 13903; individualCount: 6; occurrenceID: A24031BC-9A09-5816-8C31-916CC6B9487C; **Taxon:** family: Camaenidae; genus: Landouria; scientificNameAuthorship: (Pfeiffer, 1842); **Location:** locality: Batu Kudik, Simunjan, Sarawak

##### Distribution

Sarawak: Kuching, Samarahan and Miri Divisions (Marzuki et al. 2021). **Distribution elsewhere**: Indo-Australian archipelago (Pfeiffer 1842; Vermeulen and Whitten 1998).

##### Notes

**Figure 21.**
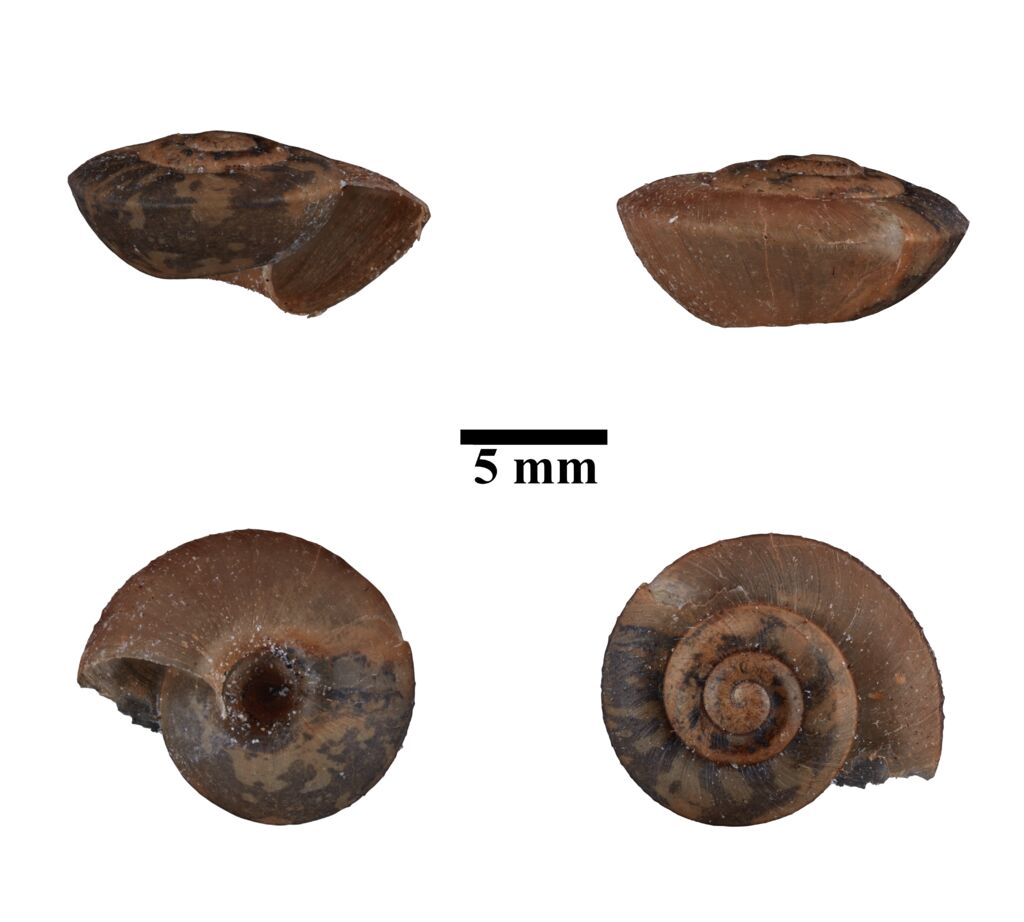
Apertural, posterial, umbilical and apical views of *Landouriawinteriana* (ME 13903). Scale = 5 mm.

Only dry shells were found during the surveys. The representative is shown in Fig. 21. The identification of the species was based on the original description by Pfeiffer (1842), species description by Marzuki et al. (2021). Neotype, not seen (ZMA 376193A).

#### Kaliella scandens

(Cox, 1871)

8A21B14C-7C62-54D5-8CDE-AE8128BDDA6E

##### Materials

- **Type status:** Other material. **Occurrence:** catalogNumber: MZU.MOL.22.453; individualCount: 3; occurrenceID: 01711D70-AFD0-5BCE-808B-3871A5491A04; **Taxon:** family: Chronidae; genus: Kaliella; scientificNameAuthorship: (Cox, 1871); **Location:** locality: Batu Kudik, Simunjan, Sarawak
- **Type status:** Other material. **Occurrence:** catalogNumber: ME 13371; individualCount: 7; occurrenceID: 6EF85010-4E63-5C49-AA76-E4A1DA725217; **Taxon:** family: Chronidae; genus: Kaliella; scientificNameAuthorship: (Cox, 1871); **Location:** locality: Batu Kudik, Simunjan, Sarawak
- **Type status:** Other material. **Occurrence:** catalogNumber: ME 13905; individualCount: 5; occurrenceID: BEB223FF-BD1C-5BE7-939A-005BB243B239; **Taxon:** family: Chronidae; genus: Kaliella; scientificNameAuthorship: (Cox, 1871); **Location:** locality: Batu Kudik, Simunjan, Sarawak

##### Distribution

Sarawak: Kuching, Serian, Samarahan and Miri Divisions (Marzuki et al. 2021). Sabah: Interior, Sandakan, Kudat, Tawau and West Coast Divisions. **Distribution elsewhere**: Indonesia: Kalimantan, South-east Asia to Australia and the Pacific Islands (Cox 1871; Vermeulen et al. 2015; Vermeulen and Liew 2022).

##### Notes

**Figure 22.**
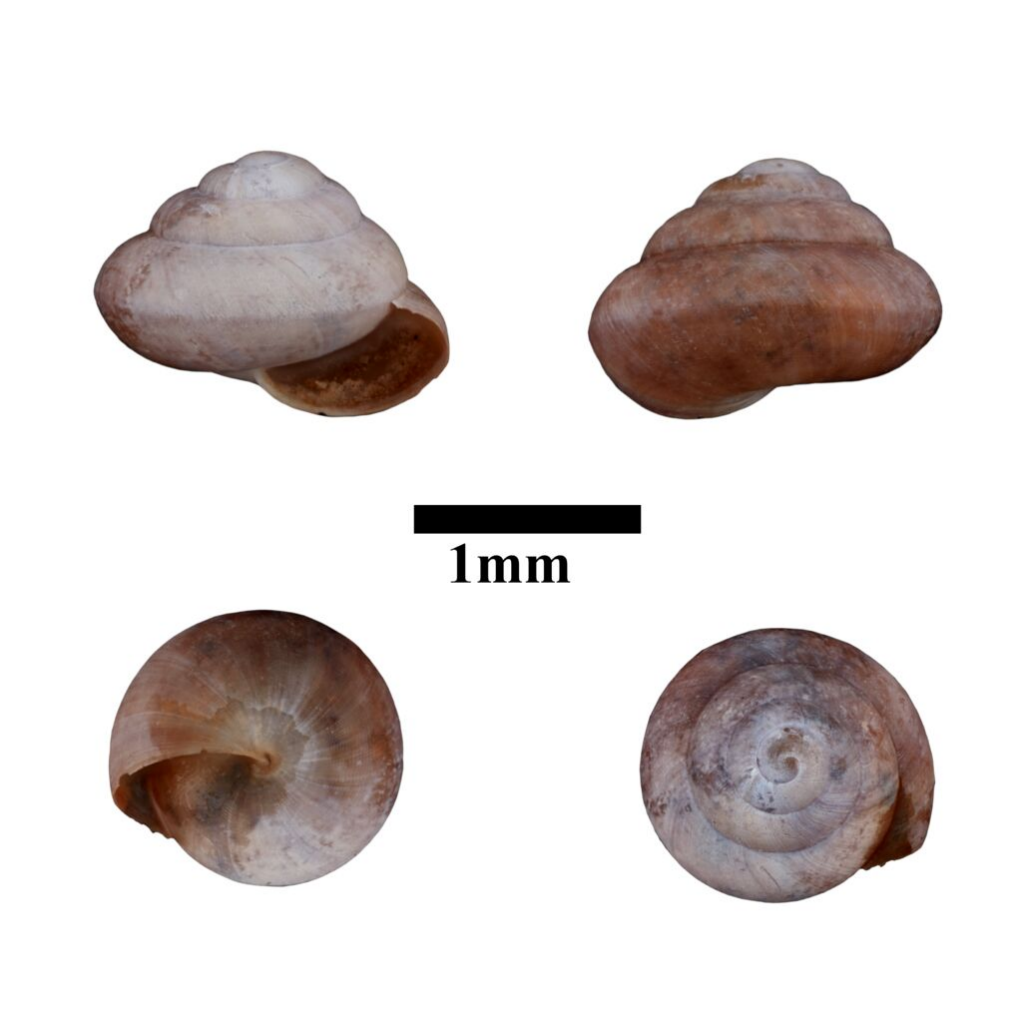
Apertural, posterial, umbilical and apical views of *Kaliellascandens* (ME 13371). Scale = 1 mm.

Living snails were observed within the arboreal area, on leaves of palms or trees at the base of the limestone cliff. The representative is shown in Fig. 22. The identification of the species was based on the original description by Cox (1871) and species description by Marzuki et al. (2021). Syntype, not seen (NHMUK 1880.12.11.17).

#### Kaliella microconus

(Mousson, 1865)

323FD011-C933-5852-9E4D-FC8A27F18BF5

##### Materials

- **Type status:** Other material. **Occurrence:** catalogNumber: MZU.MOL.22.454; individualCount: 2; occurrenceID: 530F5D89-E965-5072-91CC-AE4900E75034; **Taxon:** family: Chronidae; genus: Kaliella; scientificNameAuthorship: (Mousson, 1865); **Location:** locality: Batu Kudik, Simunjan, Sarawak
- **Type status:** Other material. **Occurrence:** catalogNumber: ME 13370; individualCount: 8; occurrenceID: E4BC73B9-BBF9-5436-B98C-B9E3417A19DC; **Taxon:** family: Chronidae; genus: Kaliella; scientificNameAuthorship: (Mousson, 1865); **Location:** locality: Batu Kudik, Simunjan, Sarawak
- **Type status:** Other material. **Occurrence:** catalogNumber: ME 13904; individualCount: 5; occurrenceID: B644A736-60E4-52A2-9701-3F4C77D03673; **Taxon:** family: Chronidae; genus: Kaliella; scientificNameAuthorship: (Mousson, 1865); **Location:** locality: Batu Kudik, Simunjan, Sarawak

##### Distribution

Sarawak: Kuching, Serian, Samarahan and Miri Divisions (Marzuki et al. 2021). Sabah: Interior, Kudat, Sandakan, Tawau and West Coast Divisions (Vermeulen and Liew 2022). **Distribution elsewhere**: Indonesia: South Kalimantan Provinces, South-east Asia to Australia and the Pacific Islands (Mousson 1865; Vermeulen and Whitten 1998).

##### Notes

**Figure 23.**
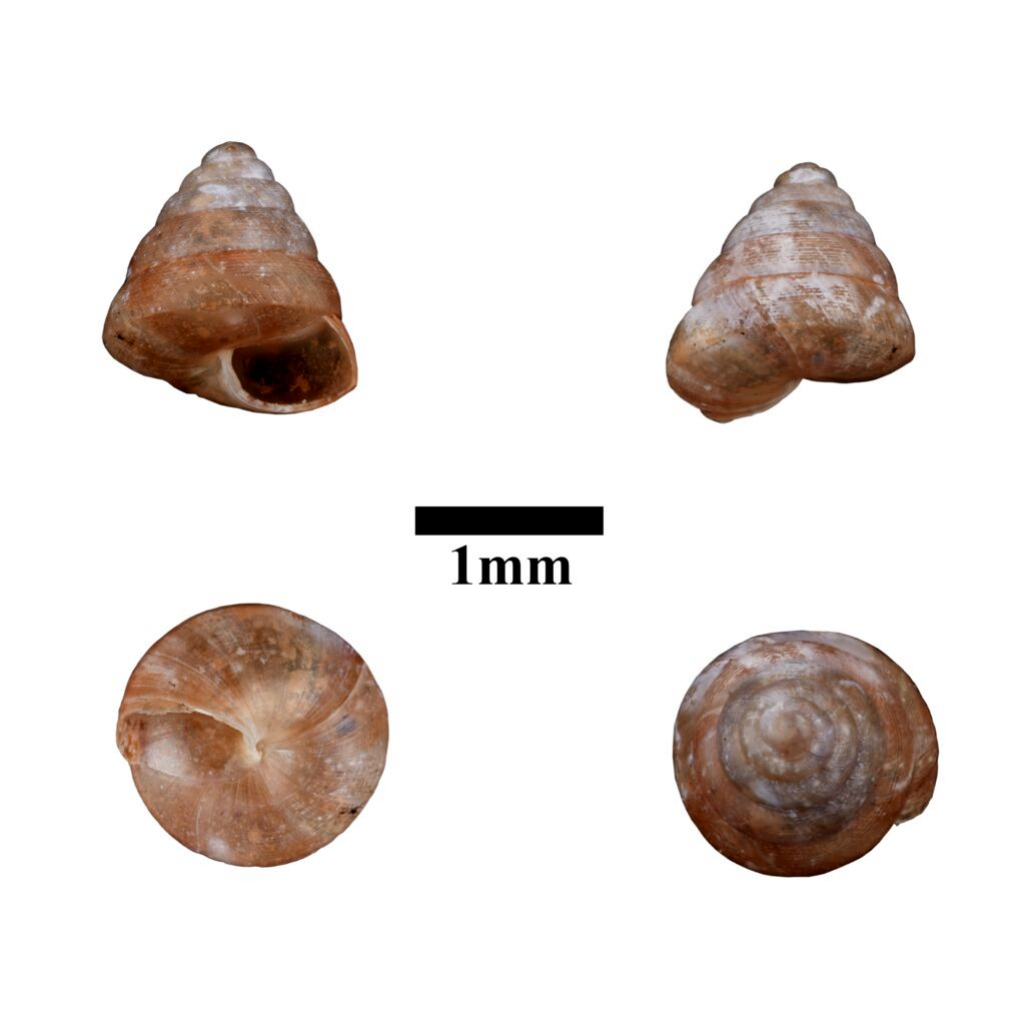
Apertural, posterial, umbilical and apical views of *Kaliellamicroconus* (ME 13370). Scale = 1 mm.

Living snails were observed amongst the leaf litter and plant debris near the limestone cliff. The representative is shown in Fig. 23. The identification of the species was based on the original description by Mousson (1865) and species description by Marzuki et al. (2021). Neotype, not seen (MNHN-IM-2000-28605).

#### Kaliella calculosa

(Gould, 1852)

6E0BA4D4-22E2-5D52-8A16-DA595D436511

##### Materials

- **Type status:** Other material. **Occurrence:** catalogNumber: MZU.MOL.22.455; individualCount: 2; occurrenceID: 0BD139EA-2937-504C-A3D7-0DF24A4643CA; **Taxon:** family: Chronidae; genus: Kaliella; scientificNameAuthorship: (Gould, 1852); **Location:** locality: Batu Kudik, Simunjan, Sarawak
- **Type status:** Other material. **Occurrence:** catalogNumber: ME 13906; individualCount: 3; occurrenceID: 388C3404-DCA5-5A0C-AB4B-3AB0EEB1E2E0; **Taxon:** family: Chronidae; genus: Kaliella; scientificNameAuthorship: (Gould, 1852); **Location:** locality: Batu Kudik, Simunjan, Sarawak

##### Distribution

Sarawak: Kuching, Serian, Samarahan and Miri Divisions (Marzuki et al. 2021). Sabah: Interior, Sandakan, Kudat, Tawau and West Coast Divisions (Vermeulen and Liew 2022). **Distribution elsewhere**: South Asia mainland to Indo-Australian archipelago and the Pacific Islands (Gould 1852; Vermeulen et al. 2015).

##### Notes

**Figure 24.**
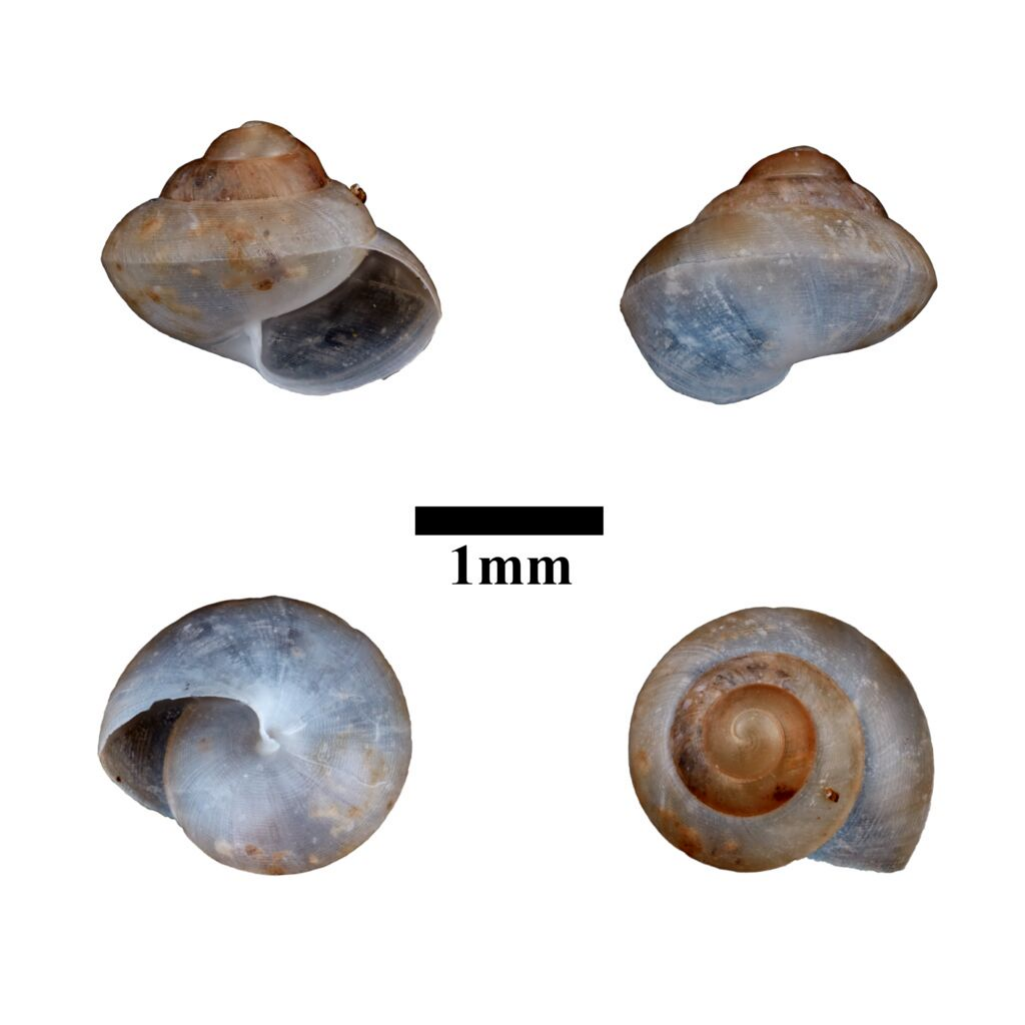
Apertural, posterial, umbilical and apical views of *Kaliellacalculosa* (ME 13906). Scale = 1 mm.

Only dry shells were found during the surveys. The representative is shown in Fig. 24. The identification of the species was based on the original description by Gould (1852) and species description by Marzuki et al. (2021). Syntype, not seen (USNM 5465).

#### Kaliella punctata

Vermeulen, Liew & Schilthuizen, 2015

8ABFABD4-37E0-57FE-9356-45B359E1410A

##### Materials

- **Type status:** Other material. **Occurrence:** catalogNumber: MZU.MOL.22.456; individualCount: 1; occurrenceID: 61D9DE36-3B7E-5672-A0CC-5B7BCE4E4072; **Taxon:** family: Chronidae; genus: Kaliella; scientificNameAuthorship: Vermeulen, Liew & Schilthuizen, 2015; **Location:** locality: Batu Kudik, Simunjan, Sarawak
- **Type status:** Other material. **Occurrence:** catalogNumber: ME 13907; individualCount: 2; occurrenceID: 572C16E5-6115-5CFA-9442-ACD18F132DA5; **Taxon:** family: Chronidae; genus: Kaliella; scientificNameAuthorship: Vermeulen, Liew & Schilthuizen, 2015; **Location:** locality: Batu Kudik, Simunjan, Sarawak

##### Distribution

Sarawak: Samarahan Division (Marzuki et al. 2021). Sabah: West Coast, Interior, Sandakan and Tawau Provinces (Vermeulen and Liew 2022). **Distribution elsewhere**: Indonesia: Kalimantan (Vermeulen et al. 2015; Vermeulen and Liew 2022). Endemic to Borneo.

##### Notes

**Figure 25.**
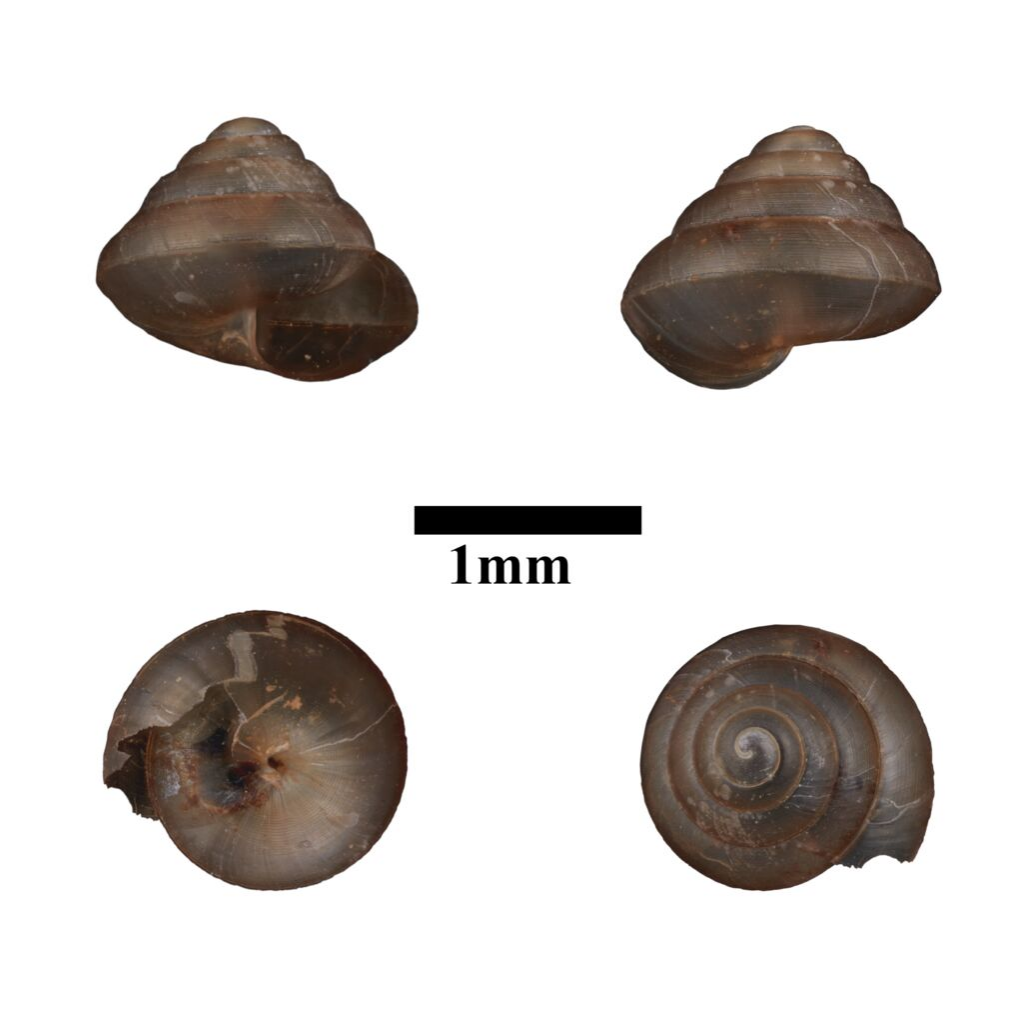
Apertural, posterial, umbilical and apical views of *Kaliellapunctata* (ME 13907). Scale = 1 mm.

Only dry shells were found during the surveys. The representative is shown in Fig. 25. The identification of the species was based on the original description by Vermeulen et al. (2015). Holotype, not seen (RMNH.5003925).

#### Everettia minuta

Marzuki, Liew & Mohd-Azlan, 2021

A1016554-5F62-5AB5-B879-990884F89AAA

##### Materials

- **Type status:** Other material. **Occurrence:** catalogNumber: ME 13372; individualCount: 1; occurrenceID: 3002C6A6-E426-5DA2-9786-115B29F1E259; **Taxon:** family: Dyakiidae; genus: Everettia; scientificNameAuthorship: Marzuki, Liew & Mohd-Azlan, 2021; **Location:** locality: Batu Kudik, Simunjan, Sarawak

##### Distribution

Sarawak: Kuching, Serian and Samarahan Divisions (Marzuki et al. 2021). Endemic to western Sarawak.

##### Notes

**Figure 26.**
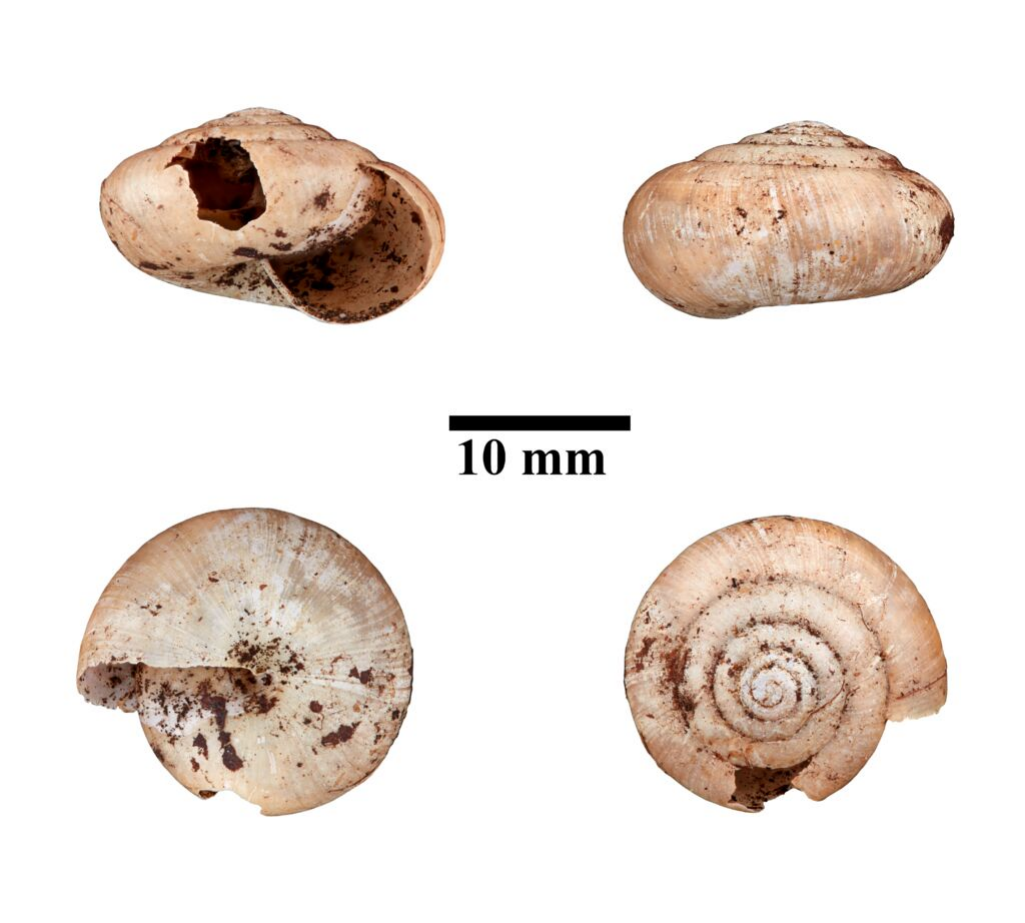
Apertural, posterial, umbilical and apical views of *Everettiaminuta* (ME 13372). Scale = 10 mm.

Only dry shells were found during the surveys. The representative is shown in Fig. 26. The identification of the species was based on the original revision by Marzuki et al. (2021). Holotype, seen (MZU.MOL.20.23).

#### Videna bicolor

(von Martens, 1864)

B632B035-7686-5B8E-84EE-D4D0A0269F68

##### Materials

- **Type status:** Other material. **Occurrence:** catalogNumber: ME 13368; individualCount: 1; occurrenceID: 5326F082-6FF2-5E94-9D62-CE2B7DD6C246; **Taxon:** family: Trochomorphidae; genus: Videna; scientificNameAuthorship: (von Martens, 1864); **Location:** locality: Batu Kudik, Simunjan, Sarawak
- **Type status:** Other material. **Occurrence:** catalogNumber: ME 13902; individualCount: 3; occurrenceID: 44E65735-68A5-5980-A3E7-457552A11050; **Taxon:** family: Trochomorphidae; genus: Videna; scientificNameAuthorship: (von Martens, 1864); **Location:** locality: Batu Kudik, Simunjan, Sarawak

##### Distribution

Sarawak: Kuching, Serian, Samarahan, Sibu, Mukah, Kapit and Miri Divisions (Marzuki et al. 2021). Sabah: Interior, Kudat, Sandakan, Tawau and West Coast Divisions (Vermeulen and Liew 2022). **Distribution elsewhere**: Indonesia: West and South Kalimantan Provinces and Sumatra to Lesser Sunda (Martens 1864; Aldrich 1889; Martens and Thiele 1908; Vermeulen and Whitten 1998).

##### Notes

**Figure 27.**
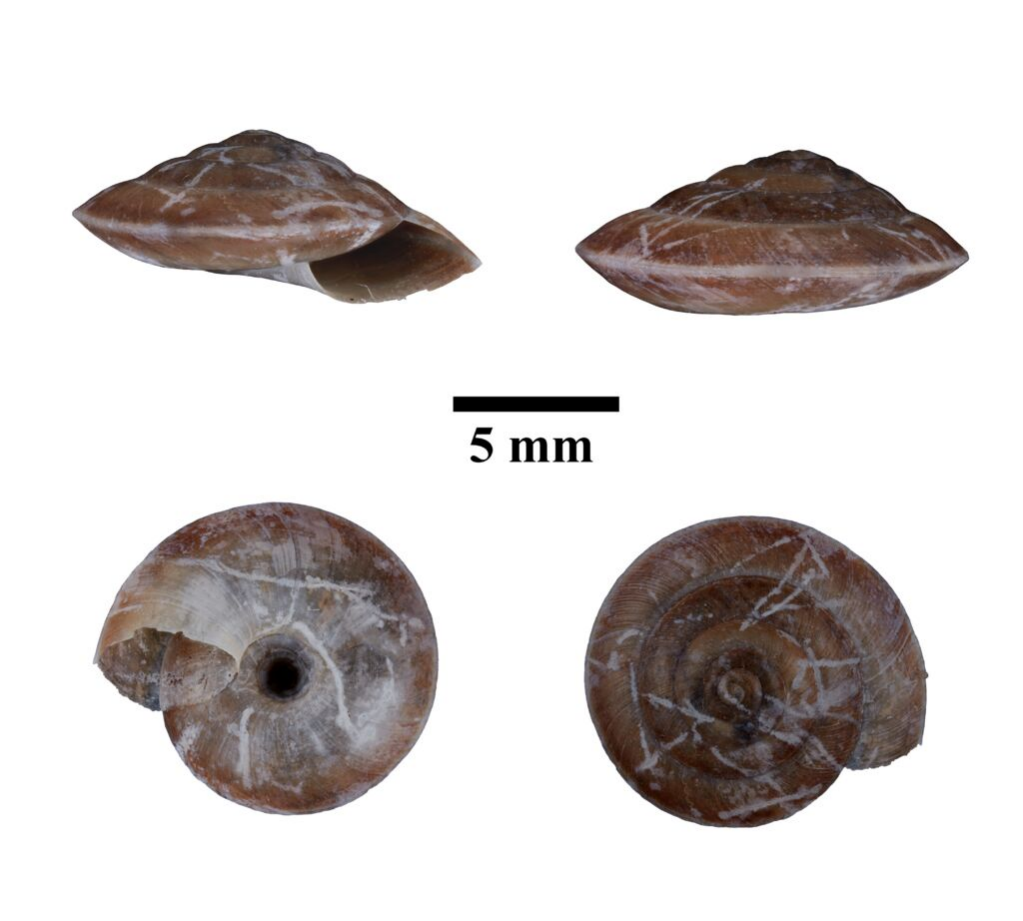
Apertural, posterial, umbilical and apical views of *Videnabicolor* (ME 13368). Scale = 5 mm.

Only dry shells were found during the surveys. The representative is shown in Fig. 27. The identification of the species was based on the original description by Martens (1864) and species description by Marzuki et al. (2021). Type specimen, not seen.

#### Philalanka kusana

(Aldrich, 1889)

05833335-572E-54AD-A285-2A4FA9C9B528

##### Materials

- **Type status:** Other material. **Occurrence:** catalogNumber: MZU.MOL.22.457; individualCount: 2; occurrenceID: B2E7F9CD-C11C-519A-88F7-C245F8676254; **Taxon:** family: Charopidae; genus: Philalanka; scientificNameAuthorship: (Aldrich, 1889); **Location:** locality: Batu Kudik, Simunjan, Sarawak
- **Type status:** Other material. **Occurrence:** catalogNumber: ME 13362; individualCount: 4; occurrenceID: 84946251-C838-532E-94C0-E80F65270C3B; **Taxon:** family: Charopidae; genus: Philalanka; scientificNameAuthorship: (Aldrich, 1889); **Location:** locality: Batu Kudik, Simunjan, Sarawak

##### Distribution

Sarawak: Kuching, Serian, Samarahan, Kapit and Miri Divisions (Marzuki et al. 2021). Sabah: Interior, Kudat, Sandakan, Tawau and West Coast Divisions (Vermeulen and Liew 2022). **Distribution elsewhere**: Indonesia: South Kalimantan Provinces and West Malaysia to Papua (Aldrich 1889; Vermeulen et al. 2015).

##### Notes

**Figure 28.**
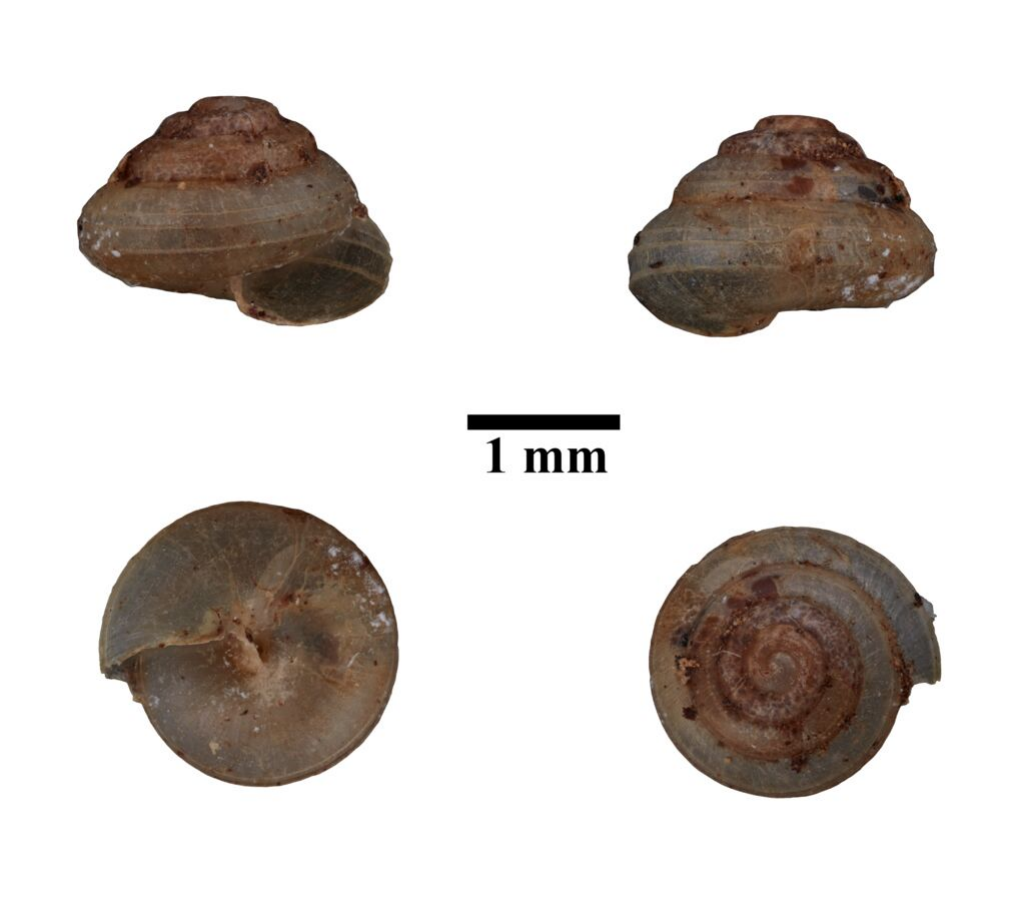
Apertural, posterial, umbilical and apical views of *Philalankakusana* (ME 13362). Scale = 1 mm.

Only dry shells were found during the surveys. The representative is shown in Fig. 28. The identification of the species was based on the original description by Aldrich (1889) and species description by Marzuki et al. (2021). Type specimen, not seen.

#### Pupisoma dioscoricola

(C. B. Adams, 1845)

68BE5300-DFE3-5144-A516-DCFA853AD2E1

##### Materials

- **Type status:** Other material. **Occurrence:** catalogNumber: MZU.MOL.22.458; individualCount: 2; occurrenceID: CB445AF8-F64D-5708-A26C-7F83917F9F4A; **Taxon:** family: Valloniidae; genus: Pupisoma; scientificNameAuthorship: (C. B. Adams, 1845); **Location:** locality: Batu Kudik, Simunjan, Sarawak
- **Type status:** Other material. **Occurrence:** catalogNumber: ME 13373; individualCount: 9; occurrenceID: A538854D-6B46-5E28-AA76-8C9CEFBDD77F; **Taxon:** family: Valloniidae; genus: Pupisoma; scientificNameAuthorship: (C. B. Adams, 1845); **Location:** locality: Batu Kudik, Simunjan, Sarawak
- **Type status:** Other material. **Occurrence:** catalogNumber: ME 13897; individualCount: 1; occurrenceID: C2AEC3FE-0107-5EBA-97A5-960FC7397570; **Taxon:** family: Valloniidae; genus: Pupisoma; scientificNameAuthorship: (C. B. Adams, 1845); **Location:** locality: Batu Kudik, Simunjan, Sarawak

##### Distribution

Sarawak: Kuching, Samarahan, Bintulu, Miri and Limbang Divisions (Marzuki et al. 2021). Sabah: West Coast, Interior, Kudat, Tawau and Sandakan Divisions (Vermeulen and Liew 2022). **Distribution elsewhere**: Africa, Asia, Australia, Pacific Islands and America (Adams 1845; Pilsbry 1921; Hausdorf 2007; Vermeulen and Liew 2022).

##### Notes

**Figure 29.**
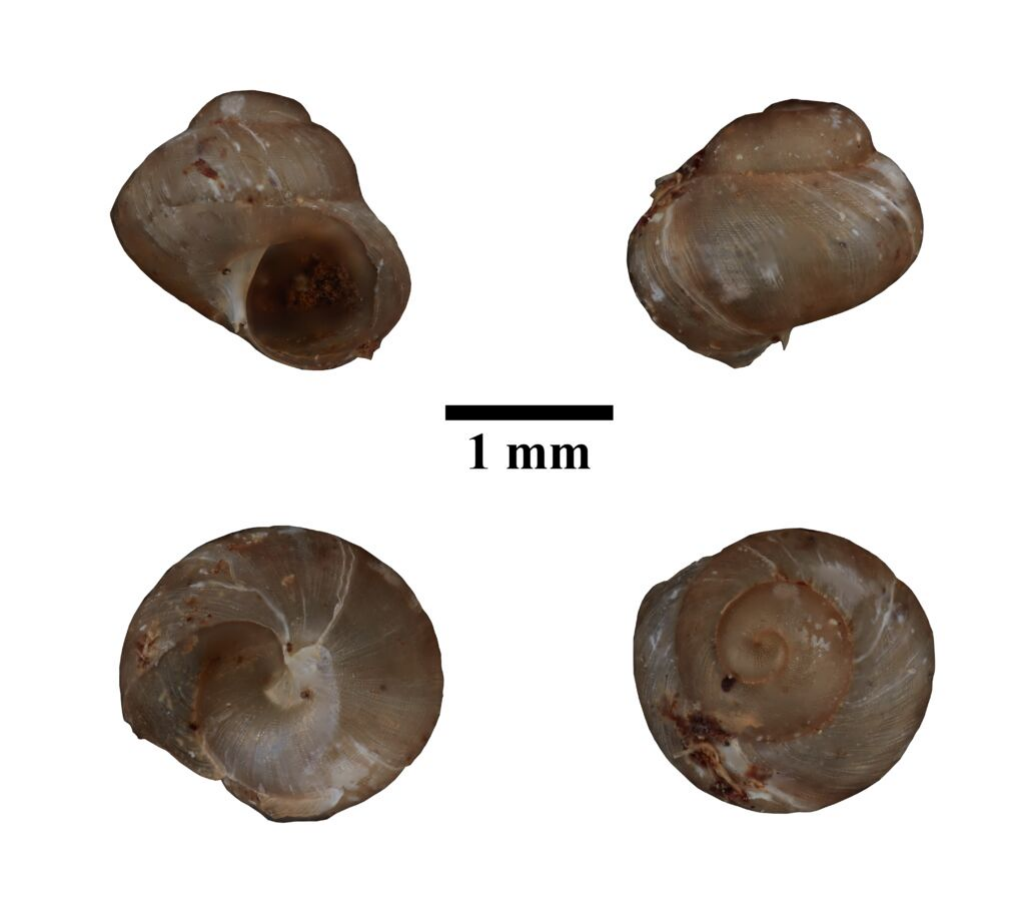
Apertural, posterial, umbilical and apical views of *Pupisomadioscoricola* (ME 13373). Scale = 1 mm.

Widely distributed. Only dry shells were found during the surveys. The representative is shown in Fig. 29. The identification of the species was based on the original description by Adams (1845). Syntype, not seen (NHMUK 1875.2.8.14).

## Analysis

A total of 24 species of land snails, representing 18 genera and 14 families were recorded (see Table 1). The family Diplommatinidae (17%) and family Chronidae (17%) are the most species-rich family with four recorded species in each family, followed by Ariophantinidae (13%), family Achatinidae (8%) and family Hydrocenidae (8%) recorded in this limestone hill cluster. *Diplommatinaconcinna*, *Diplommatinaonyx* and *Plectostomawallaceikudikense* are new subspecies belonging to the Diplommatinidae family, whereas *Kaliellascandens*, *Kaliellamicroconus*, *Kaliellacalculosa* and *Kaliellapunctata* are classified under the family Chronidae. In terms of genera, the most diverse genus within the area was *Kaliella* with four species recorded. Additionally, *Opisthostomajavanica*, *Georissapyrrhoderma* and *Plectostomawallaceikudikense* are recorded as the most abundant species. Microsnails (size less than 5 mm) accounted for ca. 63% of the total number of species, while small to medium-sized snails (size between 5 to 20 mm) accounted for ca. 37% of the total species. Surprisingly, there are only two medium-sized snails (size more than 20 mm) encountered in this survey, namely, *Everettiaminuta* and *Hemiplectadensa*.

## Discussion

During our fieldwork, it was observed that the majority of the inhabitants, including the land snails, were concentrated in the gaps between limestone boulders (Fig. 1c), rather than in the vicinity of the exposed outcrop (Fig. 1d). It is worth noting that the outcrop serves as the sole habitat for *Plectostomawallaceikudikense*. Consequently, these findings have sparked renewed conservation efforts to prevent the endemic species from becoming extinct.

The land snail fauna in Batu Kudik exhibited a lower level of endemism, with only one endemic species (4%), in comparison to the slightly larger outcrops like Bukit Sarang and the larger karst cluster in the south, which have higher endemism levels of 31.3% and 38.5% for land snails, respectively. Previous studies by Roos et al. (2004) and Clement et al. (2008) have indicated that larger karst regions tend to support more endemic species due to their greater habitat diversity, which subsequently promotes higher rates of speciation (Losos and Schluter 2000; Clement et al. 2008). This trend is also observed in other groups of organisms, such as fishes and orchids, where a positive correlation exists between the size of the area and both species richness and endemism (Clement et al. 2006; Clement et al. 2008). Additionally, the presence of only two medium-sized snails at Batu Kudik can be attributed to the fact that larger snails require more resources, such as calcium carbonate, when compared to microsnails (Goodfriend 1986; Baur and Raboud 1988). It is highly probable that the current resources available at Batu Kudik are limited, which makes it challenging for the larger snails to thrive in this environment.

Moreover, a significant portion of the limestone hills in the south (Bau Region), falls within protected areas, indicating that many of the endemic species are safeguarded. However, Batu Kudik, situated between the south and areas with agricultural activities around the limestone karst, lack such protection. As a result, factors such as quarrying, deforestation and agricultural activities become crucial determinants that may affect level of endemism, including the land snails (Schilthuizen et al. 2005; Clement et al. 2008).

## Supplementary Material

XML Treatment for Plectostoma wallacei kudikense

XML Treatment for Acmella cyrtoglyphe

XML Treatment for Japonia bellula

XML Treatment for Stomacosmethis jagori

XML Treatment for Diplommatina concinna

XML Treatment for Diplommatina onyx

XML Treatment for Opisthostoma javanica

XML Treatment for Georissa hungerfordi

XML Treatment for Georissa pyrrhoderma

XML Treatment for Allopeas gracile

XML Treatment for Allopeas clavulinum

XML Treatment for Hemiplecta densa

XML Treatment for Macrochlamys infans

XML Treatment for Microcystina paripari

XML Treatment for Helicarion dyakanum

XML Treatment for Landouria winteriana

XML Treatment for Kaliella scandens

XML Treatment for Kaliella microconus

XML Treatment for Kaliella calculosa

XML Treatment for Kaliella punctata

XML Treatment for Everettia minuta

XML Treatment for Videna bicolor

XML Treatment for Philalanka kusana

XML Treatment for Pupisoma dioscoricola

## Figures and Tables

**Figure 1a.**
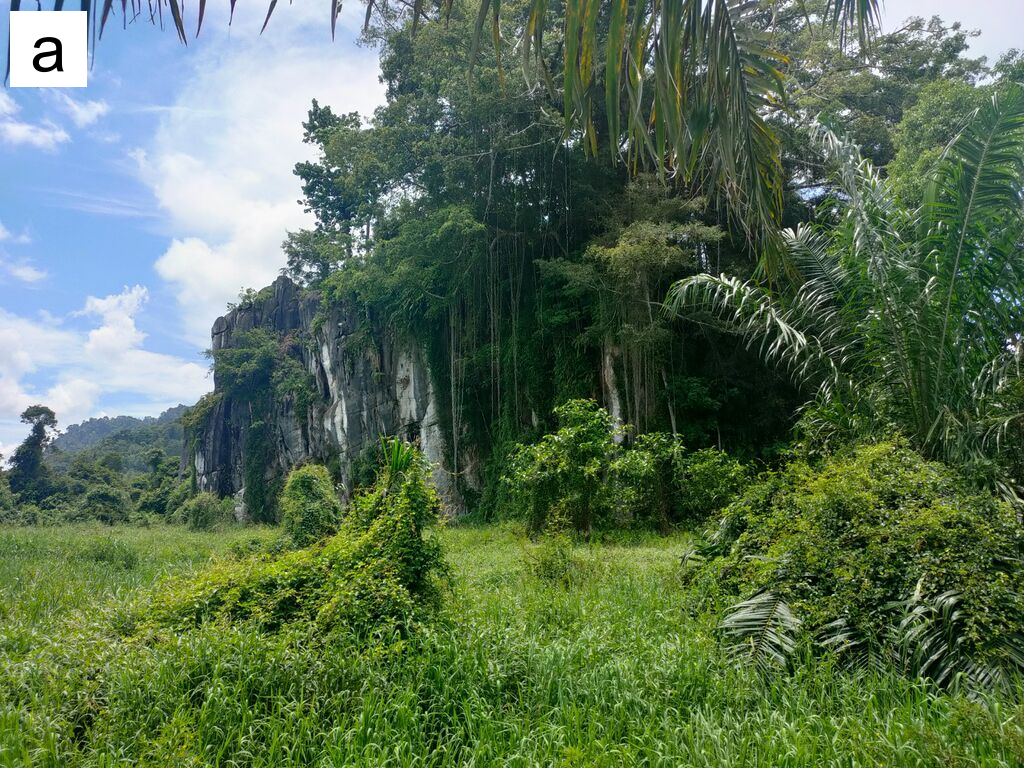
East side of the outcrop;

**Figure 1b.**
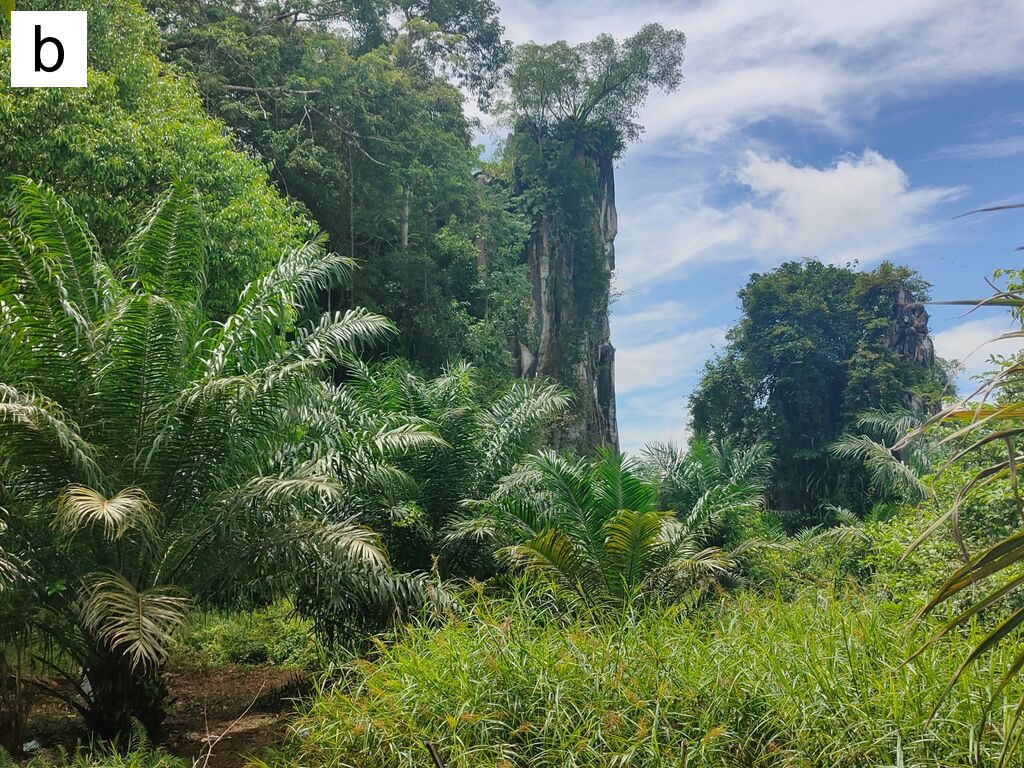
West side of the outcrop;

**Figure 1c.**
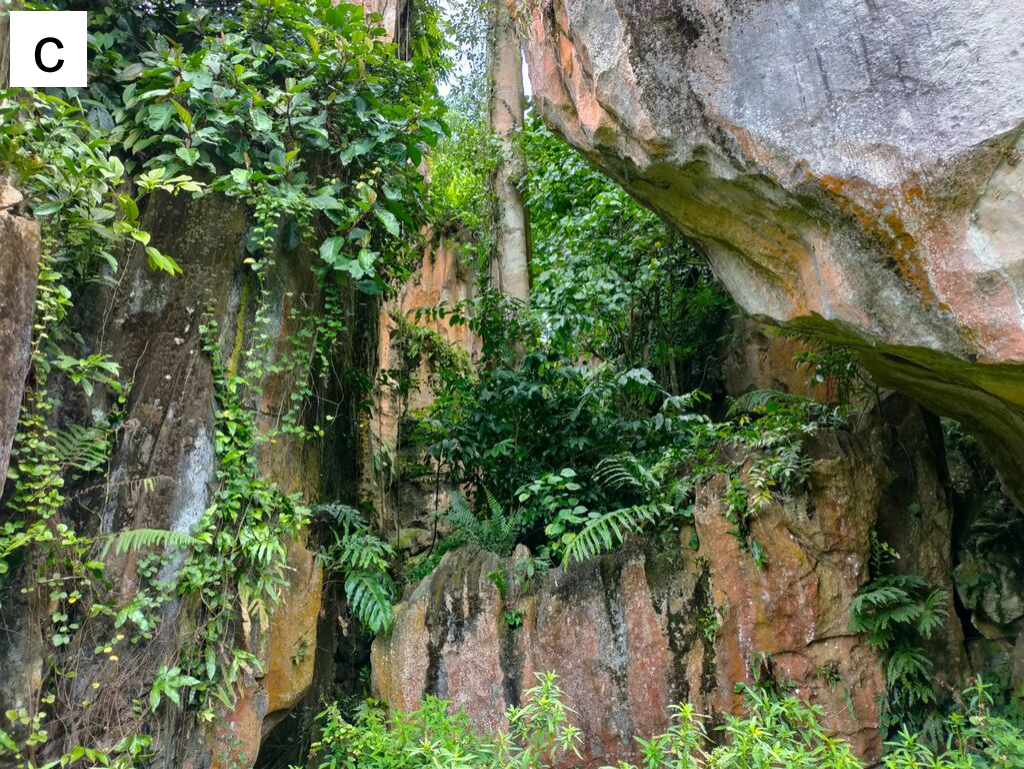
The remaining limestone inhabitants in between limestone boulders;

**Figure 1d.**
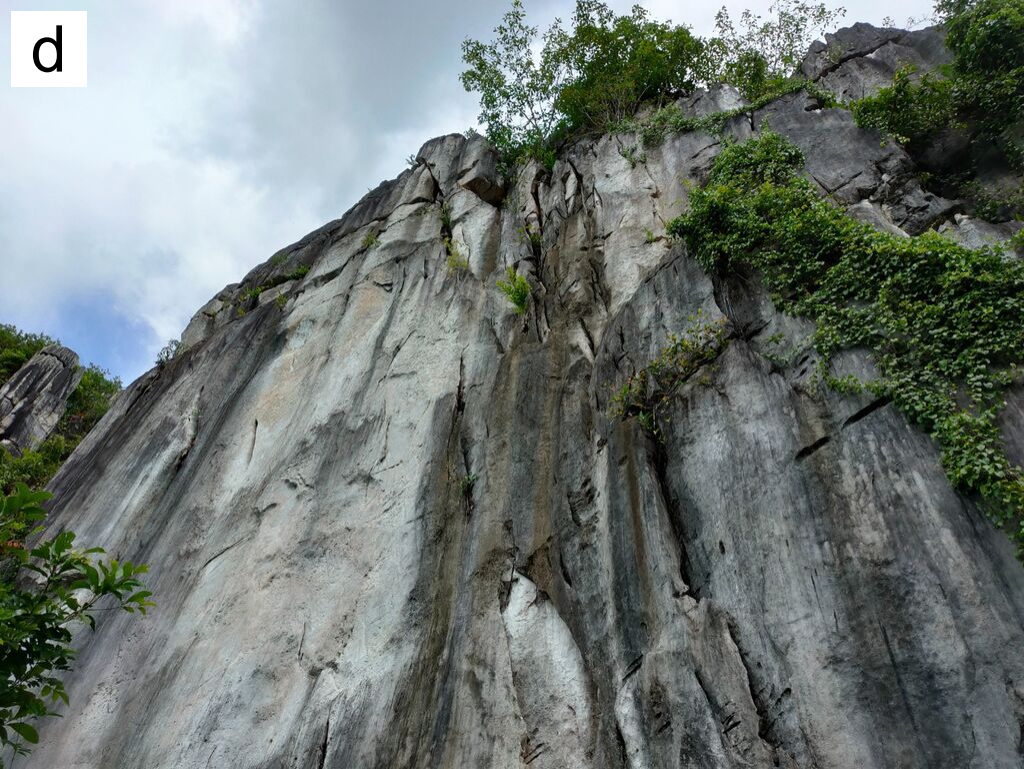
Dried, exposed outer limestone wall surfaces.

**Figure 3a.**
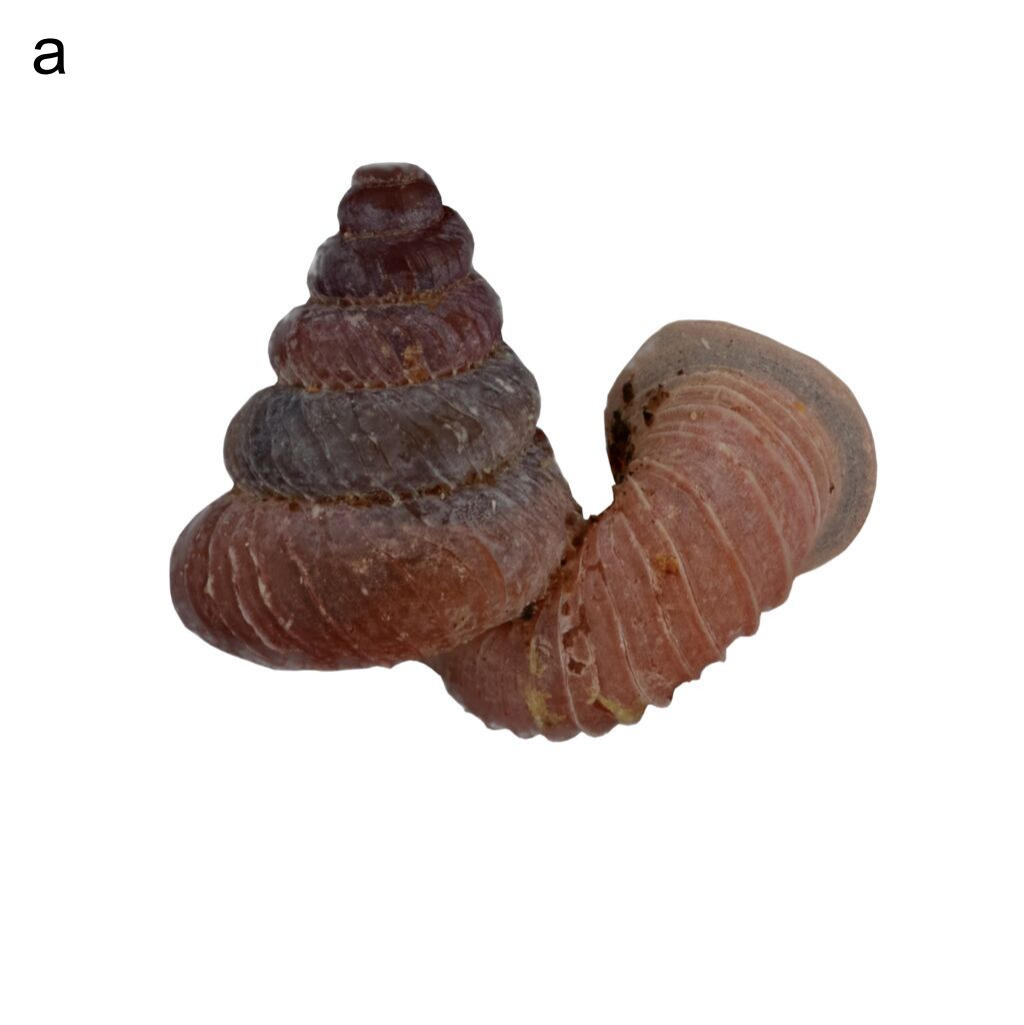


**Figure 3b.**
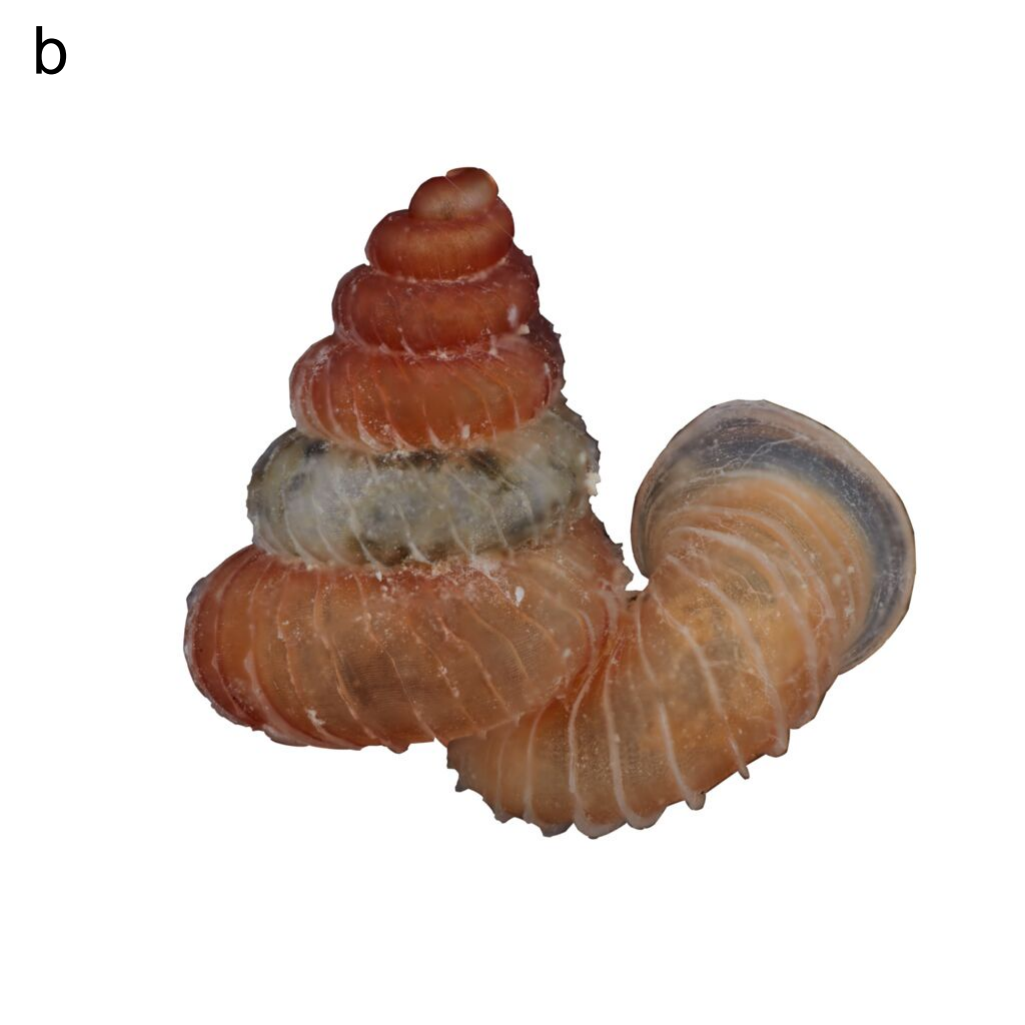


**Figure 3c.**
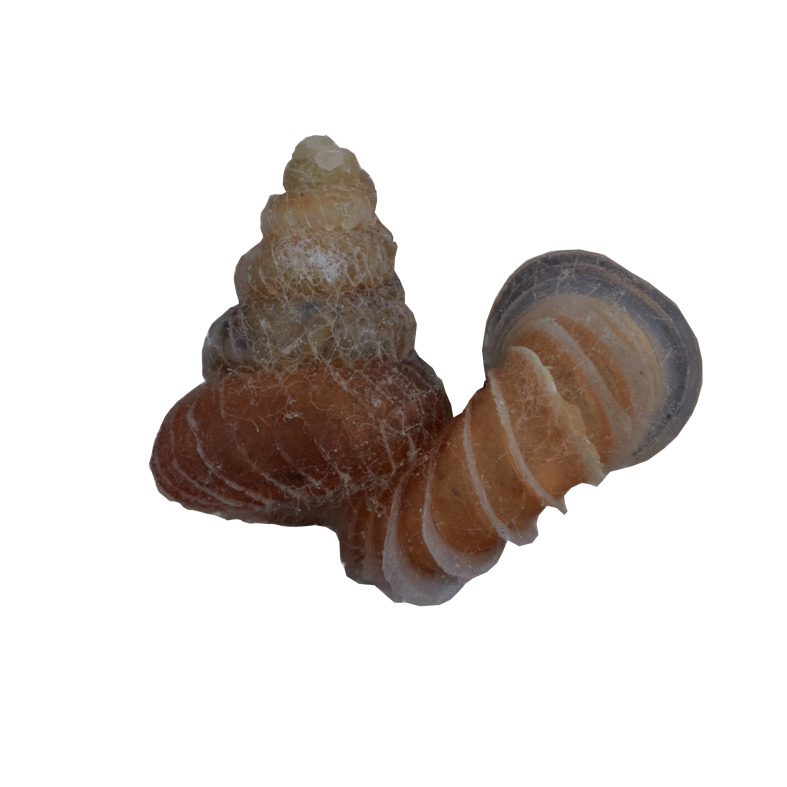


**Figure 3d.**
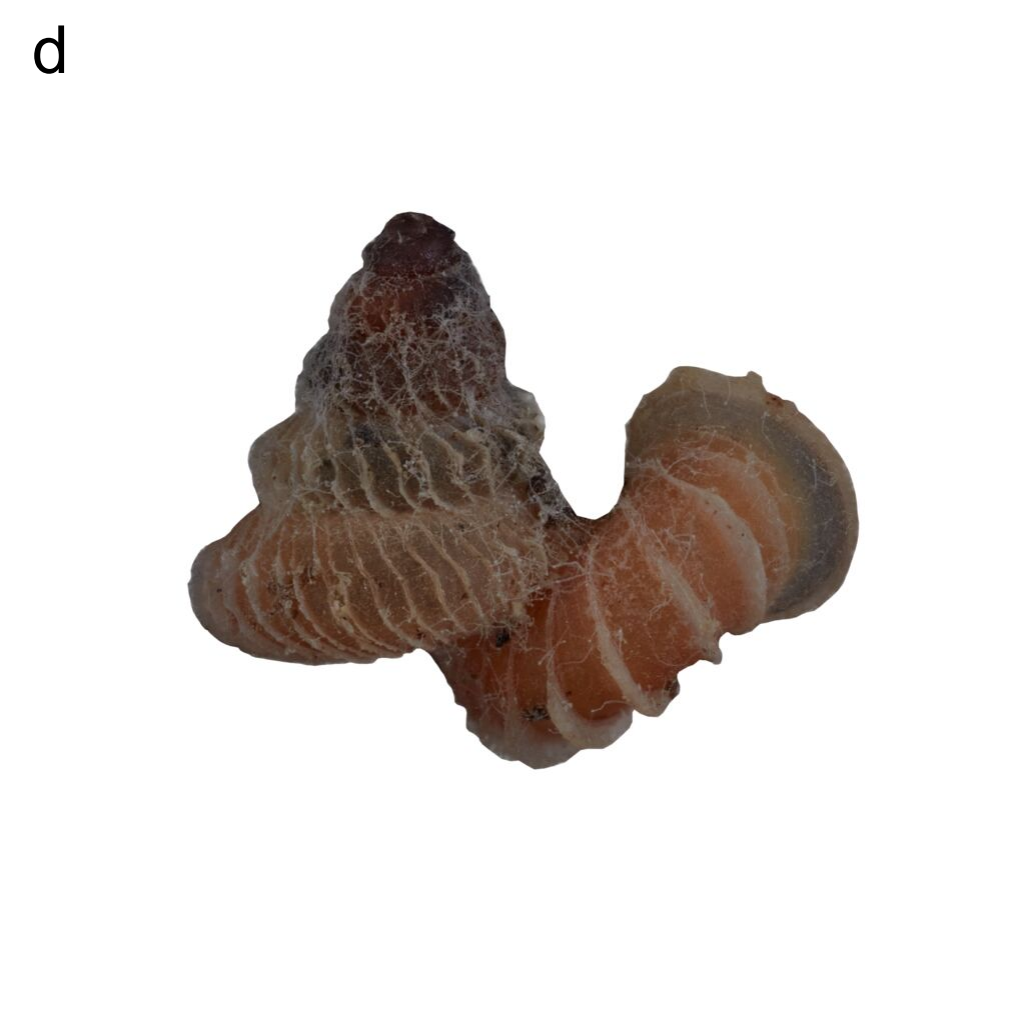


**Figure 3e.**
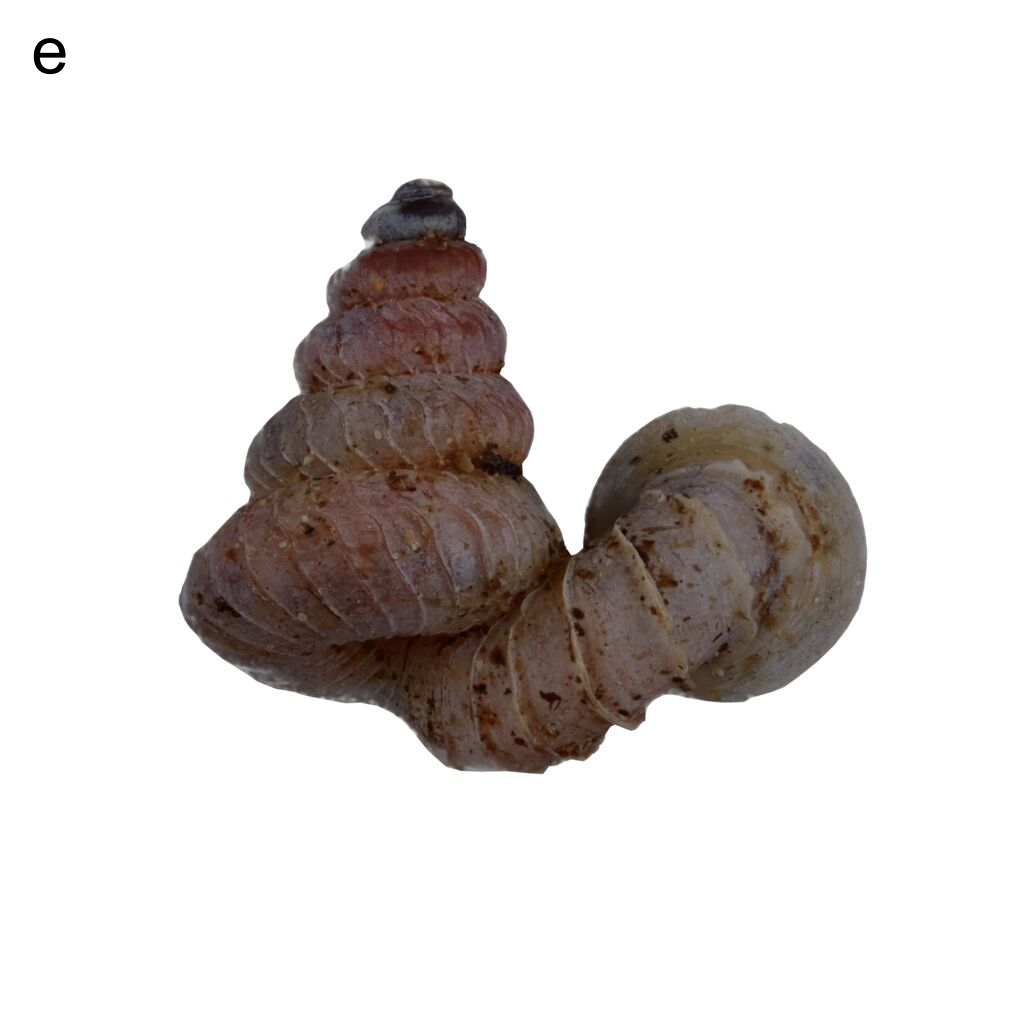


**Figure 3f.**
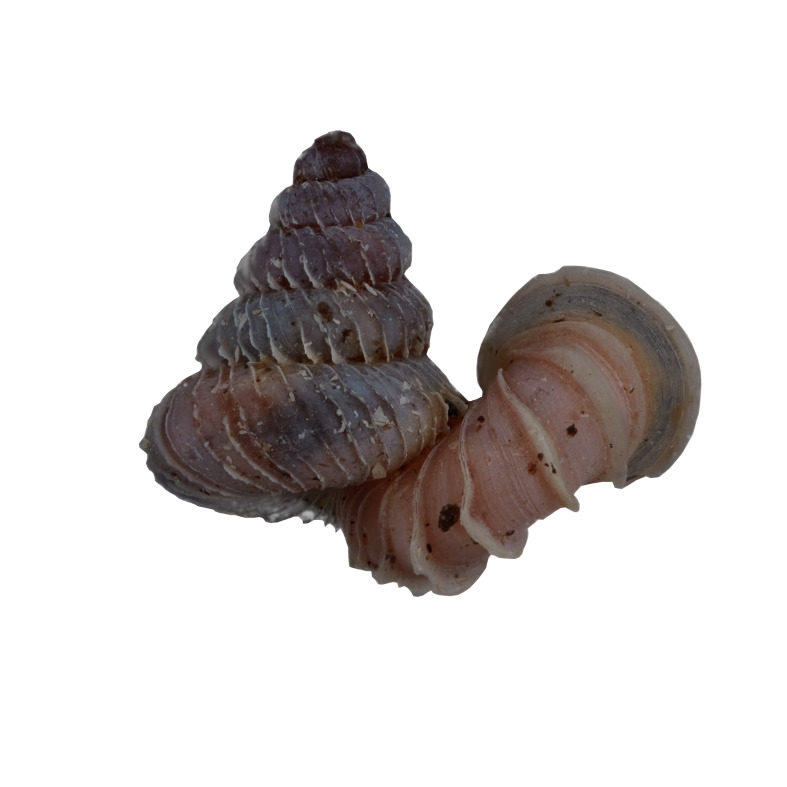


**Figure 4a.**
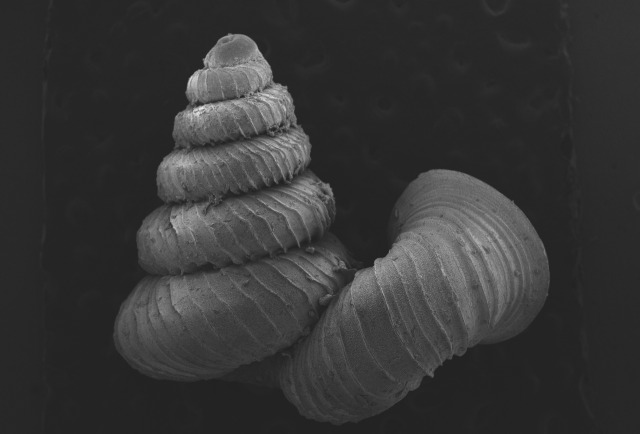
Posterior view at 30x magnification;

**Figure 4b.**
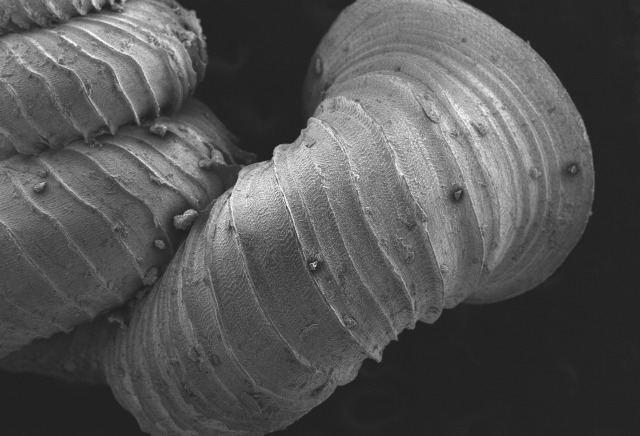
The shell tuba at 55x magnification;

**Figure 4c.**
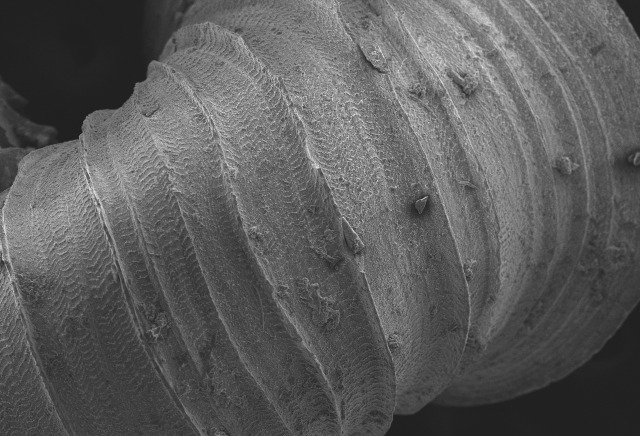
The shell tuba at 100x magnification;

**Figure 4d.**
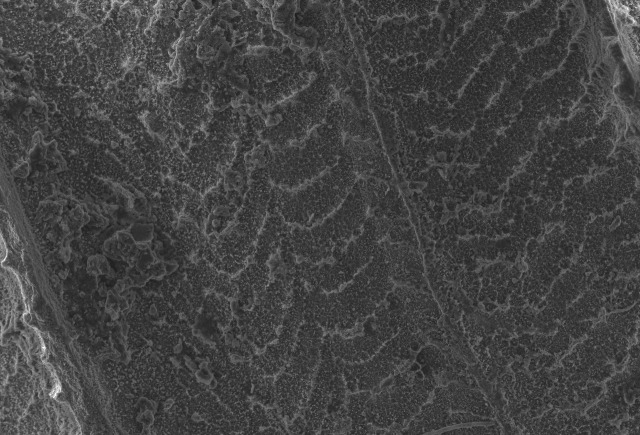
The shell tuba at 600x magnification.

**Figure 5a.**
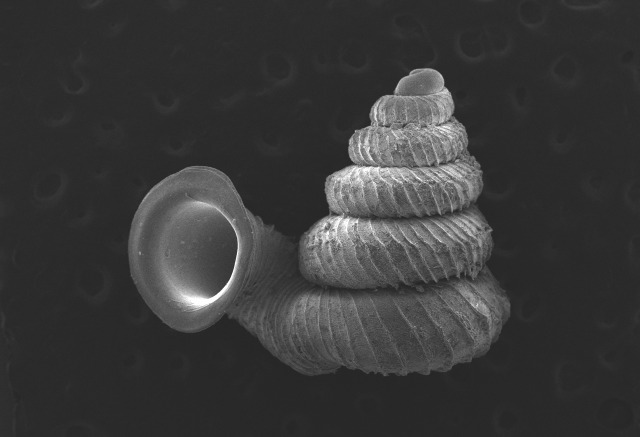
Apertural view at 30x magnification;

**Figure 5b.**
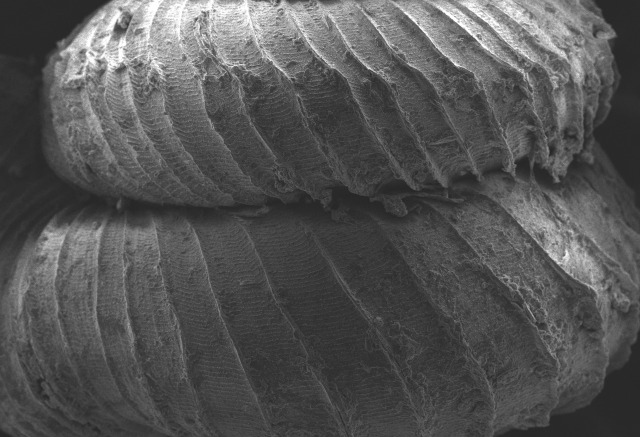
Enlargement of apertural view at 90x magnification;

**Figure 5c.**
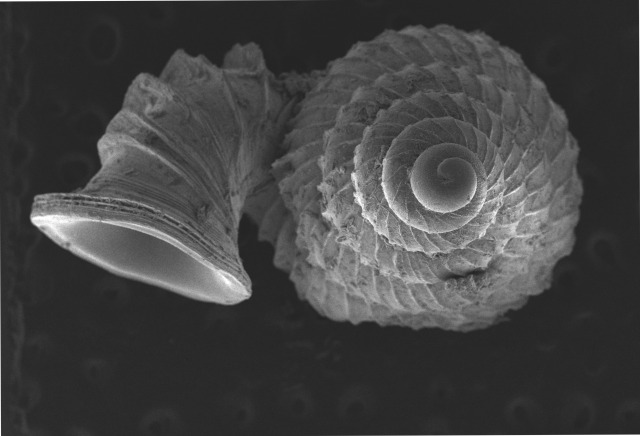
Apical view at 30x magnification;

**Figure 5d.**
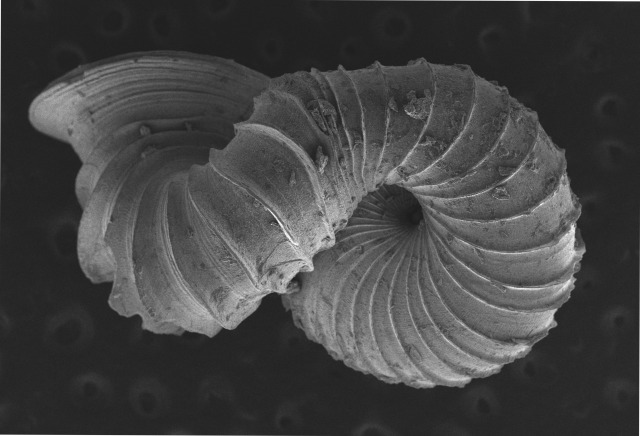
Basal view at 30x magnification.

**Table 1.** The number of individuals and the relative abundance of land snails of Batu Kudik.

Family/ Genus	Number of Individuals	Relative Species Abundance (100%)
**Achatinidae**		
*Allopeasgracile*	46	0.4887
*Allopeasclavulinum*	31	0.3293
**Alycaeidae**		
*Stomacosmethisjagori*	186	1.9760
**Assimineidae**		
*Acmellacyrtoglyphe*	3	0.0319
**Ariophantidae**		
*Hemiplectadensa*	2	0.0212
*Macrochlamysinfans*	8	0.0850
*Microcystinaparipari*	7	0.0744
**Camaenidae**		
*Landouriawinteriana*	13	0.1381
**Charopidae**		
*Philalankakusana*	6	0.0637
**Cyclophoridae**		
*Japoniabellula*	31	0.3293
**Chronidae**		
*Kaliellascandens*	15	0.1594
*Kaliellamicroconus*	15	0.1594
*Kaliellacalculosa*	5	0.0531
*Kaliellapunctata*	3	0.0319
**Diplommatinidae**		
*Diplommatinaconcinna*	552	5.8642
*Diplommatinaonyx*	1	0.0106
*Opisthostomajavanica*	3522	37.4613
*Plectostomawallaceikudikense* subsp. nov.	1828	19.4200
**Dyakiidae**		
*Everettiaminuta*	1	0.0106
**Helicarionidae**		
*Helicariondyakanum*	8	0.0850
**Hydrocenidae**		
*Georissahungerfordi*	44	0.0425
*Georissapyrrhoderma*	3070	32.6145
**Trochomorphidae**		
*Videnabicolor*	4	0.0425
**Valloniidae**		
*Pupisomadioscoricola*	12	0.1275

## References

[B10565140] Adams A., Reeve L. A. (1850). Mollusca. In: Adams A (Ed.).

[B10565148] Adams C. B. (1845). Specierum novarum conchyliorum, in Jamaica repertorum, synopsis.. Pro­ceedings of the Boston Society of Natural History.

[B10565157] Adams H. (1872). Descriptions of fourteen new species of land and marine shells.. Proceedings of the Zoological Society of London.

[B10565166] Aldrich T. H. (1889). Notes upon a collection of shells from Borneo with description of new species.. Jounal of the Cincinnati Society of Natural History.

[B10992510] Baur B., Raboud C. (1988). Life history of the land snail *Ariantaarbustorum* along an altitudinal gradient.. Journal of Animal Ecology,.

[B10983112] Beron P. (2015). Comparative study of the invertebrate cave faunas of Southeast Asia and New Guinea.. Historia naturalis bulgarica.

[B10565193] Clement R., Sodhi N. S., Schilthuizen M., Ng P. K. L. (2006). Limestone karsts of Southeast Asia: Imperiled arks of biodiversity.. BioScience.

[B10565202] Clement R., Peter N. K. L., Lu X. X., Ambu S., Schilthuizen M., Corey B. J. A. (2008). Using biogeographical patterns of endemic land snails to improve conservation planning for limestone karsts.. Biological Conservation.

[B10565213] Cox J. (1871). Description of a new volute and twelve new species of land-shells from Aus­tralia and the Solomon Islands.. Proceedings of the Zoological Society of London.

[B10565222] Fulton H. (1901). Descriptions of some supposed new species of *Diplommatina*, *Opisthostoma*, and a new variety of *Alycaeus* from N. Borneo, Banguey Islands, and Darjeeling.. The Annals and Magazine of Natural History including Zoology, Botany, and Geology.

[B10565231] Godwin-Austen H. H. (1889). On a collection of land-shells made in Borneo by Mr. A. Everett, with descriptions of supposed new species – Part I. Cyclostomacea.. Proceedings of the Zoo­logical Society of London.

[B10565240] Godwin-Austen H. H. (1891). On a collection of land-shells made in Borneo by Mr. A. Everett, with descriptions of supposed new species, part II. Zonitidae and Helicidae.. Proceedings of the Zo­ological Society of London.

[B10992501] Goodfriend G. A. (1986). Variation in land-snail shell form and size and its causes: A review.. Systematic Zoology,.

[B10565267] Gould A. A. (1852). United States Exploring Expedition during the years 1838, 1839, 1840, 1841, 1842 under the command of Charles Wilkes, U.S.N..

[B10983256] Hausdorf B. (2007). Revision of the American *Pupisoma* species (Gastropoda: Pupilloidea).. Journal of Natural History.

[B10565275] Hutton T. (1834). On the land shells of India.. The Journal of the Asiatic Society of Bengal.

[B10565284] Khalik M. Z., Hendriks K. P., Vermeulen J. J., Schilthuizen M. (2018). A molecular and conchological dissection of the “scaly” *Georissa* of Malaysian Borneo (Gastropoda, Neritimorpha, Hydrocenidae).. ZooKeys.

[B10565293] Khalik M. Z., Hendriks K. P., Vermeuleen J. J., Schilthuizen M. (2019). Conchological and molecular analysis of the “non-scaly” Bornean *Georissa* with descriptions of three new species (Gastropoda, Neritimorpha, Hydrocenidae).. ZooKeys.

[B10565324] Liew T. S., Foon J. K., Clements G. R. (2021). Conservation of limestone ecosystem of Malaysia, Part VI. Detailed information of limestone outcrops of Sarawak..

[B10565332] Losos J. N., Schluter D. (2000). Analysis of an evolutionary species-area relationship.. Nature.

[B10565341] Martens E. V. (1860). Drei neue Landschnecken..

[B10565349] Martens E. V. (1864). Diagnosen neuer Arten von Heliceen aus dem indischen Archipel..

[B10565357] Martens E. V. (1865). Über neue Landschnecken aus Ost-indien und über zwei Seesterne von Costa-Rica.

[B10565365] Martens E. V., Thiele J. (1908). Beschreibung einiger im östlichen Borneo von Dr. Martin Schmidt gesammelten Land- und Süßwasser-Conchylien..

[B10565373] Marzuki M. E., Liew T. S., Jayasilan. Mohd-Azlan (2021). Land snails and slugs of Bau limestone hills, Sarawak (Malaysia, Borneo), with the descriptions of 13 new species.. ZooKeys.

[B10565382] Mousson M. A. (1857). Novitates Zollingerianæ (1).. Journal de Conchyliologie,.

[B10565391] Mousson M. A. (1865). Coquilles terrestres et fluviatiles de quelques îles de I’Océan Pacifique, re­cueillies par M. le D’ E. Græffe.. Journal de Conchyliologie.

[B10565400] Nurinsiyah A. S., Hausdorf B. (2017). Revision of the Diplommatinidae (Gastropoda: Cyclophoroidea) from Java.. Zootaxa.

[B10565409] Pfeiffer L. (1842). Symbolae ad historiam heliceorum, sectio alterna..

[B10565417] Pilsbry H. A. (1921). Manual of conchology, structural and systematic, 2nd Series: with illustrations of the species: Pulmonata, vol. 26. Pupillidae (Vertigininae, Pupillinae)..

[B10565434] Potiez V. L. V., Michaud A. L. G. (1838). Galerie des mollusques, ou catalogue méthodique, descrip­tive et raisonné des mollusques et coquilles du Muséum de Douai..

[B10565442] Reeve L. A. (1854). Monograph of the genus *Helix*. In: Reeve LA (Ed.).

[B10565450] Roos M. C., Keßler P. J. A., Gradstein S. R., Baas P. (2004). Species diversity and edemism of five major Malesian islands: diversity-area relationships.. Journal of Biogeography.

[B10565467] Schilthuizen M., Thor-Seng L., Elahan B., Lackman-Ancenaz I. (2005). Effects of karst forest degradation on pulmonate and prosobranch land snail communities in Sabah, Malaysian Borneo.. Conservation Biology.

[B10565476] Smith E. A. (1895). On a collection of land-shells from Sarawak, British North Borneo, Palawan, and other neighbouring islands.. Proceedings of the Zoological Society of London.

[B10565485] Smith E. A. (1899). A list of the land-shells of the island of Lombock, with descriptions of new species.. Proceedings of the Malacological Society of London.

[B10565494] Team R. RStudio: Integrated Development Environment for R. Boston, MA.. http://www.rstudio.com/.

[B10565502] Thompson F. G., Dance S. P. (1983). Non-marine mollusks of Borneo. II Pulmonata: Pupillidae, Clausiliidae. III Prosobranchia: Hydrocenidae, Helicinidae.. Bulletin of the Florida State Museum Biological Sciences.

[B10620734] van Benthem-Jutting T (1932). Notes on land Mollusca of the Malay Archipelago. Journal of Conchology.

[B10565511] Vermeulen J. J. (1991). Notes on the non-marine molluscs of the island of Borneo 2. The ge­nus *Opisthostoma* (Gastropoda
Prosobranchia: Diplommatinidae).. Basteria.

[B10565520] Vermeulen J. J. (1993). Notes on the non-marine mollusks of the island of Borneo 5. The genus *Diplommatina* (Gastropoda
Prosobranchia: Diplommatinidae).. Basteria.

[B10992519] Vermeulen J. J., Whitten J. A. (1998). Fauna Malesiana guide to the land snails of Bali..

[B10815161] Vermeulen J. J, Whitten T (1999). Biodiversity and cultural property in the management of limestone resources : lessons from East Asia.

[B10565546] Vermeulen J. J., Junau D. J. (2007). Bukit Sarang (Sarawak, Malaysia), an isolated limestone hill with an extraordinary snail fauna.. Basteria.

[B10565555] Vermeulen J. J., Liew T. S., Schilthuizen M. (2015). Additions to the knowledge of the land snails of Sabah (Malaysia, Borneo), including 48 new species.. ZooKeys.

[B10565564] Vermeulen J. J., Liew T. S. (2022). Land snails and slugs of Sabah and Labuan (Malaysia)..

